# A TCER-1-siRNA regulatory axis suppresses antibacterial innate immunity in *C. elegans*

**DOI:** 10.1371/journal.ppat.1013972

**Published:** 2026-07-28

**Authors:** Nikki Naim, Francis R.G. Amrit, Mayur N. Devare, Guled A. Osman, Laura L. Bahr, Hannah Henry, Brooke E. Montgomery, Spencer M. Kuhn, Taiowa A. Montgomery, Arjumand Ghazi

**Affiliations:** 1 Department of Pediatrics, University of Pittsburgh School of Medicine, John G. Rangos Sr. Research Center, One Children’s Hospital Drive, Pittsburgh, Pennsylvania, United States of America; 2 Department of Biology, Colorado State University, Fort Collins, Colorado, United States of America; 3 Department of Cell Biology and Physiology, University of Pittsburgh School of Medicine, Pittsburgh, Pennsylvania, United States of America; University of California San Diego, UNITED STATES OF AMERICA

## Abstract

Small interfering RNAs (siRNAs) are important regulators of gene expression with well-established roles in pathogen defense. Yet, their specific roles in antibacterial immunity are not well understood. Here, we identify an siRNA pathway involved in repressing antibacterial innate immunity in *Caenorhabditis elegans*. We show that genes required for the biogenesis or function of WAGO Argonaute-associated siRNAs, called 22G-RNAs, function in a common genetic pathway with the immune-suppressive transcription elongation and splicing factor TCER-1 to inhibit immunity. Loss of *tcer-1* led to reduced levels of 22G-RNAs from a subset of WAGO targets, while mutations in several WAGO 22G-RNA pathway genes phenocopied the enhanced immunoresistance of *tcer-1* mutants, suggesting a shared regulatory module. Integrative 22G-RNA-mRNA analyses and molecular genetic studies show that this module does not induce widespread gene silencing, but instead targets a restricted set of immune-relevant effectors, including *scrm-4*, which encodes a conserved phospholipid translocase that promotes host resistance. Together, our findings establish endogenous WAGO 22G-RNAs as repressors of antibacterial immunity and identify TCER-1 as a physiological regulator that promotes 22G-RNA biogenesis to constrain host defense. The results uncover a previously unrecognized small RNA-dependent mechanism linking transcription, metabolism, and antibacterial innate immunity.

## Author summary

Small interfering RNAs (siRNAs) are recognized as having critical roles in controlling how genes and proteins involved in diverse biological processes are expressed, including during immune response following viral infections. But, their role in immunity against bacterial pathogens is not clear. We provide evidence for an siRNA pathway, regulated by the *C. elegans* protein TCER-1, that represses antibacterial innate immunity. TCER-1, along with WAGO Argonaute proteins involved in 22G-RNA biogenesis, promotes the production of a subset of 22G-RNAs that repress a select group of targets, including a lipid-modifying scramblase enzyme, SCRM-4, that is necessary for immune activation. This results reveals a novel small RNA-dependent mechanism of antibacterial innate immunity.

## Introduction

Non-coding small RNAs, including microRNAs (miRNAs) and siRNAs, mediate RNA interference (RNAi)-based gene silencing to regulate diverse processes ranging from development to genome defense [1–5]. miRNAs are widely established as conserved regulators of innate and adaptive immune responses against viral and bacterial pathogens, whereas siRNAs are known to have roles in antiviral immunity [1,4–8]. In plants and invertebrates, Dicer-mediated processing of viral double-stranded RNA and subsequent Argonaute-dependent silencing constitute a canonical, genetically well-defined antiviral pathway [8–11]. In mammals, miRNAs have well-defined roles in shaping innate and adaptive immune programs [1,7], whereas functions for endogenous siRNAs remain poorly defined [12–15]. Similarly, although small RNAs contribute to antibacterial responses in plants and invertebrates, these functions have largely been described in the context of miRNAs [16–19].

The nematode *Caenorhabditis elegans*, despite lacking professional immune cells, deploys defense programs regulated by conserved signaling cascades [20]. miRNA-based antibacterial defenses have been described in *C. elegans*, along with roles for specific Argonaute proteins [19,21–26]. In *C. elegans*, endogenous siRNAs are dominated by two branches defined by their Argonaute interactors: CSR-1 and the WAGOs, which together comprise several distinct proteins [27]. The CSR-1 22G-RNA branch licenses germline gene expression, whereas the WAGO 22G-RNA branch mediates exogenous and endogenous RNAi, including repression of aberrant transcripts and transposons [3]. Previous work has revealed roles for the piwi-interacting RNA (piRNA) and downstream RNAi pathway in bacterial sensing and pathogen-avoidance learning [28–30]. Recently, CSR-1 was shown to promote antibacterial resistance [21], but how distinct Argonaute-associated small RNA pathways contribute to antibacterial immunity remains unclear.

We previously identified TCER-1, the *C. elegans* homolog of human transcription elongation and splicing factor TCERG1 [31–35], as a repressor of host defense against multiple Gram-positive and Gram-negative pathogens, as well as abiotic stressors [36]. *tcer-1* mutants exhibit enhanced immunoresistance, whereas its overexpression shortens post-infection survival [36]. Mutants of the *Arabidopsis thaliana* TCER-1 homolog, PRP40, also show increased pathogen resistance [37]. TCER-1 has emerged in genetic screens for RNAi regulators suggesting that it may suppress immunity via a small RNA pathway [38,39]. Here, we show that *tcer-1* mutants exhibited reduced levels of 22G-RNAs from a subset of WAGO targets, and mutations in several WAGO genes phenocopied the enhanced immunoresistance of *tcer-1* mutants against the human opportunistic pathogen *Pseudomonas aeruginosa* strain PA14 (PA14). Integrative small RNA (sRNA)-mRNA sequencing revealed that this pathway does not induce global gene silencing but targets select immune-relevant effectors, including *scrm-4*, encoding a conserved phospholipid translocase, that promotes host defense. Together, our findings establish endogenous WAGO 22G-RNAs as repressors of antibacterial immunity and identify TCER-1 as a physiological regulator that promotes 22G-RNA biogenesis to constrain host defense.

## Results

### Overlapping roles for TCER-1, PPW-1, and RRF-1 in suppressing anti-bacterial immunity

**Fig 1:**
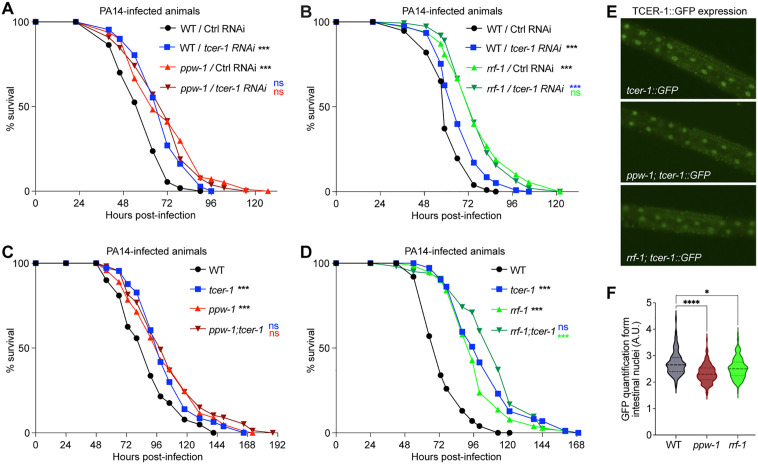
TCER-1, RRF-1, and PPW-1 act non-additively to regulate antibacterial immunity. **(A-D)** Impact of *tcer-1* inactivation by RNAi (A, B) or mutation (C, D) on survival of wildtype (WT) animals and *ppw-1* or *rrf-1* mutants following PA14 exposure. **A, B:** WT, *ppw-1(pk1425)* and *rrf-1(ok589)* grown from egg to L4 larval stage on bacteria expressing dsRNA targeting the control empty vector (Ctrl) or *tcer-1* and exposed to PA14 from L4 onwards. **A:** WT, Ctrl (black: m = 58.52 ± 1.48, n = 63/81); WT, *tcer-1* RNAi (blue, m = 69.23 ± 1.48, n = 78/109); *ppw-1* Ctrl RNAi (red, m = 71.2 ± 1.95, n = 99/112), *ppw-1*, *tcer-1* RNAi (maroon, m = 70.14 ± 1.62, n = 110/121). **B:** WT, Ctrl RNAi (black, m = 60.93 ± 0.95, n = 110/134); WT, *tcer-1* RNAi (blue, m = 67.91 ± 1.09, n = 129/157); *rrf-1*, Ctrl RNAi (neon green, m = 77.45 ± 1.67, n = 114/136); *rrf-1*, *tcer-1* RNAi (olive green, m = 77.3 ± 1.33, n = 117/154). **C-D:** Survival of WT and mutant strains upon PA14 exposure from L4 stage onwards. **C:** WT (black, m = 88.97 ± 2.01, n = 116/143), *tcer-1(tm1452)* (blue, m = 102.76 ± 2.3, n = 96/111), *ppw-1(pk1425)* (red, m = 102.95 ± 2.38, n = 134/148), *ppw-1(pk1425);tcer-1(tm1452)* (maroon, m = 106.68 ± 2.94, n = 95/116). **D:** WT (black, m = 74.9 ± 1.34, n = 108/130), *tcer-1(tm1452)* (blue, m = 103.91 ± 2.45, n = 91/120), *rrf-1(ok589)* (neon green, m = 97.99 ± 1.89, n = 113/137), *rrf-1(ok589);tcer-1(tm1452)* (olive green, m = 108.5 ± 2.55, n = 88/106). **E, F:** Representative images (E) and quantification (F) of TCER-1::GFP expression in intestinal nuclei of WT animals and *ppw-1(pk1425)* and *rrf-1(ok589)* mutant strains. In A-D, mean survival following PA14 exposure shown in hours (m) ± SEM; n = observed/total (see Methods for details). Significance was calculated and using the log-rank method (Mantel Cox, OASIS2), and *p* values were adjusted for multiple testing using the Bonferroni procedure. Statistical significance shown on each panel with the color of the asterisk indicating the strain being compared to. *p* < 0.05 (*), < 0.01 (**), < 0.001 (***), not significant (ns). Data from additional trials are presented in S1 and S2 Tables.

TCER-1 is broadly expressed in the soma and germline [36,40]. To identify the tissues in which TCER-1 is necessary to repress immunity, we used *ppw-1(pk1425)* and *rrf-1(ok589)* mutants, which restrict RNAi to the soma and germline, respectively. *ppw-1* encodes an Argonaute and *rrf-1* encodes an RNA-directed RNA polymerase; both genes function specifically within the WAGO 22G-RNA pathway and strains containing mutations in these genes are widely used for tissue-restricted RNAi in worms [41,42]. Unexpectedly, both *ppw-1* and *rrf-1* mutants survived significantly longer upon PA14 infection compared to wildtype animals (Fig 1A and 1B and S1 Table). Moreover, *tcer-1* RNAi did not further enhance post-infection survival in either mutant (Fig 1A and 1B and S1 Table). Additionally, mutating *ppw-1* or *rrf-1* did not significantly further extend post-infection survival in *tcer-1(tm1452)* mutants, suggesting that PPW-1 and RRF-1 may regulate immunity through the same pathway as TCER-1 (Fig 1C and 1D and S2 Table). Given their roles in gene regulation, we tested whether *tcer-1*, *rrf-1*, or *ppw-1* regulate each other’s expression as a potential explanation for their shared functions in PA14 resistance. GFP expression from a TCER-1::GFP translational reporter [36,40] was modestly reduced in the intestinal nuclei of *ppw-1* and *rrf-1* mutants, suggesting that TCER-1 may act downstream of these genes (Fig 1E and 1F). However, *rrf-1* mRNA levels were modestly reduced in *tcer-1* mutants (~1.5-fold) based on the RNA-seq analysis described below, suggesting a positive feedback loop or a more complex regulatory hierarchy between *tcer-1, ppw-1* and *rrf-1*.

### Multiple WAGO 22G-RNA biogenesis factors suppress immunoresistance

**Fig 2:**
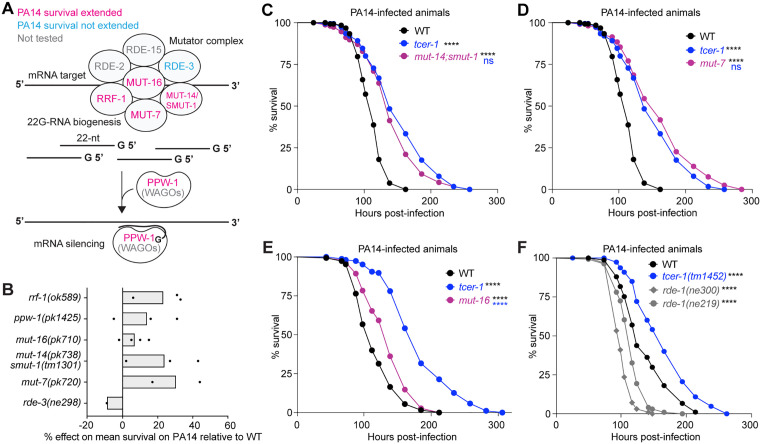
Loss of WAGO 22G-RNA biogenesis genes leads to enhanced immunoresistance. **A:** Schematic of the WAGO 22G-RNA pathway. **B:** Percent effect of 22G-RNA biogenesis factor mutations on survival upon PA14 infection compared to WT control (black line). Each data points represents percent effect in an independent trial. **C-F:** Representative graphs showing survival of *mut-14 smut-1* **(C)**, *mut-7* **(D)**, *mut-16* (E) and *rde-1* (F) mutants following PA14 infection compared to WT animals and *tcer-1* mutants. **C:** WT (m = 111.5 ± 1.9, n = 112/162), *tcer-1(tm1452)* (m = 151.4 ± 4.2, n = 120/201), *mut-14(pk738) smut-1(tm1301)* (m = 141.8 ± 3.7, n = 134/160). **D:** WT (m = 111.5 ± 1.9, n = 112/162), *tcer-1(tm1452)* (m = 151.4 ± 4.2, n = 120/201), *mut-7(pk720)* (m = 160.9 ± 5.5, n = 89/115). **E:** WT (m = 118.2 ± 2.7, n = 137/155), *tcer-1(tm1452)* (m = 183.11 ± 4.3, n = 119/170), *mut-16(pk710)* (m = 135.95 ± 2.9, n = 127/145). **F:** WT (m = 137.64 ± 3.3, n = 117/158), *tcer-1(tm1452)* (m = 166.53 ± 4.4, n= 94/167), *rde-1(ne300)* (m = 102.26 ± 1.2, n= 105/134), *rde-1(ne219)* (m = 115.25 ± 2.0, n = 105/135). In C-F, survival following PA14 exposure is shown in mean hours (m) ± SEM; n = observed/total (see Methods for details). Significance was calculated and using the log-rank method (Mantel Cox) and *P* values were adjusted for multiple testing using the Bonferroni procedure. Statistical significance shown on each panel with the color of the asterisk indicating the strain being compared to. *p* < 0.0001 (***), not significant (ns). Data from additional trials and other details are in S3 Table.

To further explore the role of 22G-RNAs in the PA14 response, we examined resistance in several additional WAGO pathway mutants - *mut-16*, *mut-14,* and *smut-1* (which function partially redundantly), *mut-7*, and *rde-3* - all of which encode core components of the Mutator complex (Fig 2A) [43–48]). With the exception of *rde-3*, all exhibited enhanced PA14 resistance comparable to *tcer-1* mutants, although *mut-16* showed a more modest effect across trials (Fig 2B and 2E and S3 Table). RDE-3 is a nucleotidyltransferase that promotes entry of cleaved mRNAs into the 22G-RNA pathway via poly(UG) tailing [49]. The lack of enhanced survival of *rde-3* mutants suggests that this activity may not be required for suppressing antibacterial resistance, or that it also performs functions that offset this effect. In contrast to WAGO 22G-RNA factors, mutations in *rde-1*, which encodes an Argonaute required for exogenous 22G-RNA production and antiviral RNAi, but which is largely dispensable for endogenous 22G-RNAs [50–57], reduced survival on PA14 relative to wildtype (Fig 2F and S3 Table). This indicates that the WAGO 22G-RNA pathway’s role in regulating antibacterial resistance is distinct from its role in the antiviral RNAi pathway involving RDE-1.

### *tcer-1* is required for normal levels of 22G-RNAs from a subset of WAGO targets

**Fig 3:**
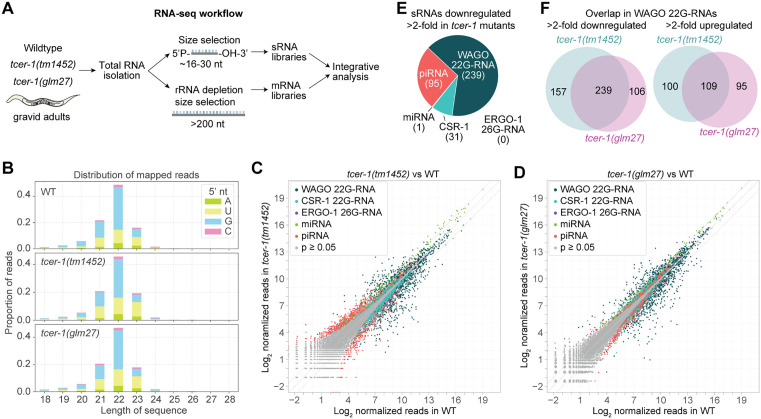
*tcer-1* inactivation leads to depletion of a subset of WAGO 22G-RNAs. **A:** Schematic of tandem sRNA-mRNA-seq pipeline. **B:** Size and 5’-nt distribution of sRNA-seq reads in WT and *tcer-1* mutants. One of 3 biological replicates is shown for each strain. **C-D:** Scatterplots showing individual small RNA-producing genes as the average log_2_ geometric mean (GM)-normalized sRNA-seq reads in WT animals (x axis) and *tcer-1(tm1452)* (C) or *tcer-1(glm27)* mutants (D) (y axis). *p* values were calculated using the Wald test with DESeq2. Small RNAs are color-coded by class. **E:** Pie chart showing the classification of the 366 small RNA features reduced in both *tcer-1* mutants. **F:** Overlap of WAGO targets showing >2-fold enrichment or depletion of 22G-RNAs in *tcer-1* mutants. See S4 Table for differential expression analysis of sRNAs.

Based on our observations above and prior reports implicating TCER-1 in the WAGO 22G-RNA pathway [38,39], we asked whether TCER-1 is required for the formation or accumulation of WAGO 22G-RNAs and whether it also plays a role in regulating WAGO target mRNAs. To address these questions, we performed high-throughput sequencing of small RNAs and mRNA from developmentally synchronized, 72-hour adult *tcer-1(tm1452)* mutants and wildtype animals grown on nonpathogenic *Escherichia coli* (OP50) (Fig 3A). Because *tcer-1(tm1452)* mutants exhibit developmental asynchrony that can potentially confound RNA-seq analysis due to stage and tissue specific gene expression [58], we included an independently generated CRISPR deleted allele, *tcer-1(glm27)*, which phenocopies *tcer-1(tm1452)*- including enhanced PA14 resistance- but does not show developmental asynchrony (S1 Fig).

Global small RNA profiles were highly similar between wildtype and *tcer-1* mutants, and were dominated by 22-nucleotide (nt), 5’G-containing sequencing reads characteristic of 22G-RNAs [52], indicating that *tcer-1* does not broadly dictate the small RNA landscape (Fig 3B). Small RNAs were classified as miRNAs, piRNAs, or siRNAs, with siRNAs subclassified by their associated Argonautes (CSR-1, WAGO, or ERGO-1) and grouped by the gene from which they were processed [3]. Both *tcer-1* alleles produced highly concordant effects on small RNA abundance (Fig 3C and 3D and S4 Table). A total of 366 small RNA features were reduced in both mutants, 270 (74%) of which were 22G-RNAs. Among these 270 that affected 22G-RNA loci, 31 (11%) corresponded to CSR-1 targets, whereas 239 (89%) were WAGO targets (Fig 3E and S4 Table). Of the 2,215 annotated WAGO targets analyzed [45], 348 were misexpressed in *tcer-1* mutants, including the 239 that were downregulated and an additional 109 that were upregulated in both *tcer-1* alleles (Fig 3F and S4 Table). These data indicate that TCER-1 selectively influences 22G-RNA formation or stability from a subset of WAGO targets. Although these effects may be indirect, possibly caused by altered processing of WAGO target mRNAs in *tcer-1* mutants, our genetic epistasis analyses are consistent with a model in which TCER-1 and WAGO 22G-RNAs function through a shared set of targets to suppress immunity.

### TCER-1 functions through the 22G-RNA target *scrm-4* to repress antibacterial resistance

**Fig 4:**
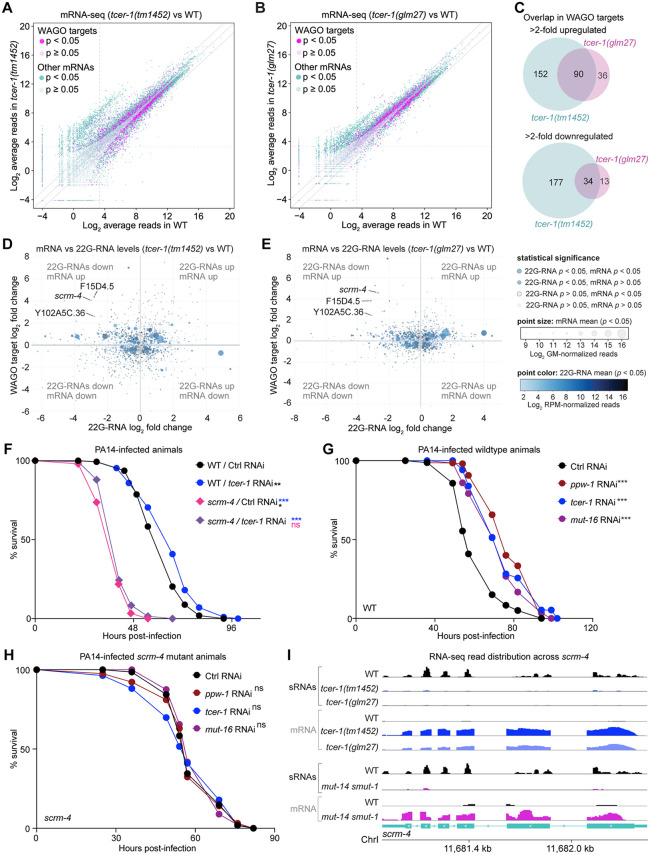
Mutation of the TCER-1 and WAGO 22G-RNA target *scrm-4* compromises antibacterial immunity. **A-B:** Scatterplots showing WAGO 22G-RNA producing genes as the average log_2_ GM-normalized mRNA-seq reads in wildtype (WT, x axis) and *tcer-1(tm1452)* (A) or *tcer-1(glm27)* mutants **(B)** (y axis). n = 3 biological replicates. **C:** Overlap in WAGO 22G-RNA producing genes enriched or depleted of mRNA-seq reads >2-fold in *tcer-1* mutants. See S5 Table for differential expression analysis of all mRNA features. **D-E:** Correlation between WAGO 22G-RNAs and target mRNAs. Cosmic plots displaying WAGO 22G-RNA producing genes as a function of log_2_ fold change in mRNA levels (y axis) and 22G-RNA levels (x axis) in *tcer-1(tm1452)* (D) or *tcer-1(glm27)* mutants **(E)**. Read abundance and statistical significance are indicated as outlined in the key in **(E)**. **F-H:** Survival of WT and *scrm-4(ok3596)* mutants grown from egg to L4 larval stage on empty vector control (Ctrl) or *tcer-1* **(F)** or *ppw-1* **(G, H)** RNAi-inducing bacteria and subsequently exposed to PA14. **F:** WT/Ctrl RNAi (m = 62.42 ± 1.0, n = 118/150), WT/*tcer-1* RNAi (m = 66.42 ± 1.22, n = 112/157), *scrm-4*/Ctrl RNAi (pink, m = 39.29 ± 0.57, n = 141/149), *scrm-4*/*tcer-1* RNAi (purple, m = 41.86 ± 0.56, n = 144/150). **G:** Survival of WT animals on Ctrl RNAi (black, m = 64.75 ± 1.7, n = 61/89), *tcer-1* RNAi (blue, m = 80.82 ± 2.11, n = 39/90), *ppw-1* RNAi (red, m = 81.8 ± 2.01, n = 30/80), *mut-16* RNAi (plum, m = 78.85 ± 2.2, n = 30/93). **H:** Survival of *scrm-4* mutants on Ctrl RNAi (black, m = 61.78 ± 1.43, n = 52/74), *tcer-1* RNAi (blue, m = 60.3 ± 1.7, n = 56/82), *ppw-1* RNAi (red, m = 60.03 ± 1.48, n = 64/81), *mut-16* RNAi (plum, m = 63.9 ± 1.42, n = 45/82). **I:** sRNA and mRNA read distribution across *scrm-4* in WT and *tcer-1* and WT and *mut-14 smut-1* mutant adult animals. One of three biological replicates is shown. Reads normalized by million reads. In F-H, survival is shown in mean hours (m) ± SEM; n = observed/total (see Methods for details). Significance was calculated using the log-rank method (Mantel Cox) and *P* values were adjusted for multiple testing using the Bonferroni procedure. Statistical significance shown on each panel with the color of the asterisk indicating the strain being compared to. *p* < 0.0001 (***), *p* < 0.001 (**), *p* < 0.05 (*), not significant (ns). Data from additional trials and other details shown in S8 Table.

Using the mRNA-seq data generated in parallel with the sRNA-seq data, we assessed the impact of *tcer-1* loss on mRNA expression of WAGO 22G-RNA pathway genes [45,46]. None of the known genes involved in WAGO 22G-RNA biogenesis or amplification were significantly downregulated >2-fold in both *tcer-1* mutants, indicating that misexpression of WAGO pathway genes is unlikely to underlie the observed loss of 22G-RNAs. However, more modest changes, such as an ~ 1.3-fold downregulation of *rrf-1*, could contribute to the phenotype (S5 Table). Both *tcer-1* mutants exhibited modest, bidirectional changes in WAGO target gene expression; of the 2,215 annotated WAGO targets only a small fraction were consistently misexpressed in both mutants, with 90 upregulated and 34 downregulated (Fig 4A and 4C and S5 Table).

Given the disparity between the numbers of misregulated 22G-RNAs and WAGO target mRNAs in *tcer-1* mutants, we next assessed the relationship between WAGO target mRNA levels and corresponding 22G-RNA abundance. There was no clear global anticorrelation between the mRNA and 22G-RNA levels, consistent with similarly weak anticorrelation in *mut-16*, *wago-1*, and *wago-3* mutants in prior reports (Fig 4D and 4E and S6 and S7 Tables) [59,60]. Only 14 WAGO targets exhibited the expected anticorrelative relationship between 22G-RNA and mRNA abundance and had both 22G-RNAs and mRNAs that were misexpressed >2-fold in both *tcer-1* mutants (S6 and S7 Tables). Among these, only three genes, *F15D4.5*, *Y102A5C.36*, *scrm-4*, showed particularly strong effects, with 22G-RNAs reduced >4-fold and corresponding mRNA levels increased by >4-fold (Fig 4D and 4E and S6 and S7 Tables). While *F15D4.5* and *Y102A5C.36* encode poorly characterized proteins lacking conserved domains or obvious functional annotations, *scrm-4* encodes a phospholipid scramblase orthologous to human PLSCR3, a protein implicated in membrane phospholipid translocation, mitochondrial structure and function, and apoptosis [61–63]. Lipid metabolism is a well-established regulator of *C. elegans* immunity [64], and our recent work demonstrated that TCER-1 promotes phospholipid remodeling to shape immunometabolism [65]. Together, the (i) strong regulatory signature, (ii) functional annotation and evolutionary conservation, and (iii) direct biological relevance to TCER-1-mediated immune regulation led us to ask whether *scrm-4* contributed to the PA14 response. *scrm-4* loss-of-function mutants survived significantly shorter that wildtype upon PA14 exposure (Fig 4F). Moreover, *tcer-1* RNAi failed to extend survival on PA14 in *scrm-4* mutants, but did in wildtype, suggesting that *tcer-1* functions through *scrm-4* to control PA14 response (Fig 4F). RNAi knockdown of *ppw-1* and *mut-16* in *scrm-4* mutants also did not extend post-infection survival, unlike in wildtype animals, consistent with a common function (Fig 4G and 4H and S8 Table). Further, changes in *scrm-4* mRNA and 22G-RNA levels were similar in *tcer-1* and *mut-14 smut-1* mutants (Fig 4I), suggesting a shared role for TCER-1 and the WAGO 22G-RNA pathway in regulating *scrm-4* expression.

Our results indicate that *scrm-4* is regulated through the activities of both *ppw-1* and *rrf-1*. Although *ppw-1* is specifically required for RNAi in the germline and *rrf-1* for RNAi in the soma, both genes are expressed in somatic and germline tissues [24,46]. Additionally, *scrm-4* expression was low but similar across mRNA-seq datasets from whole animals and dissected distal gonads, a pattern consistent with other genes whose expression is not restricted to either somatic or germline tissues, such as *tcer-1* (S2A Fig) [59]. *scrm-4* has a promoter region marked by H3K4me3 and accessible chromatin by ATAC-seq, consistent with transcription at the locus (S2B Fig). However, the surrounding genomic region is enriched for H3K27me3, a histone modification associated with transcriptional repression, and shows relatively low H3K36me3, a mark of active transcription, indicating that *scrm-4* resides within a repressive chromatin environment poised for regulated silencing (S2B Fig) [66]. *scrm-4* is likely regulated by 22G-RNAs at both the transcriptional and post-transcriptional levels, as 22G-RNAs mapping to this locus associate with the broadly expressed somatic Argonautes, PPW-1, PPW-2, and WAGO-1, as well as the germline nuclear Argonaute HRDE-1, which promotes H3K9 trimethylation and transcriptional silencing (S2C Fig) [24,67]. Further studies are needed to determine precisely where and how the WAGO 22G-RNA pathway regulates *scrm-4*, however, our results suggest that TCER-1 works with this pathway to downregulate *scrm-4* and suppress antibacterial immunity.

## Discussion

In this study, we identified WAGO 22G-RNAs as suppressors of antibacterial immunity in *C. elegans*. Our results suggest that the WAGO Argonaute PPW-1 modulates this response, consistent with a recent report that *ppw-1* and *sago-2* mutants show enhanced survival 72 hours after exposure to PA14 [24]. Our efforts to assess a genetic interaction between *tcer-1* and *sago-2* gave equivocal results across trials (S9 Table). Given the functional overlap amongst WAGO Argonautes [24,52,68], it is possible that additional WAGOs function in this pathway as well. We showed that the WAGO 22G-RNA pathway acts within an immune-suppressive regulatory program involving the transcription elongation and splicing factor TCER-1. Furthermore, our integrated sRNA- and mRNA-sequencing, together with *in vivo* molecular genetic analyses, suggest that TCER-1 and the WAGO pathway regulate immunity through selective targeting of specific, immunologically relevant genes rather than through broad siRNA-mediated silencing. One such target is *scrm-4*, a lipid-metabolic gene that contributes to host defense in a TCER-1-dependent manner.

The piRNA pathway promotes *P. aeruginosa* avoidance in *C. elegans*, thereby protecting animals from pathogen infection [28–30]. In contrast, our results indicate that the WAGO 22G-RNA pathway has the opposite effect on pathogen susceptibility by suppressing immunoresistance. While the piRNA and WAGO 22G-RNA pathways intersect, they likely have distinct roles in pathogen avoidance and susceptibility, since the small RNAs reduced in *tcer-1* mutants are predominantly WAGO 22G-RNAs, whereas those depleted following PA14 exposure are largely piRNAs. Furthermore, loss of the piRNA biogenesis factor *prde-1* did not enhance survival on PA14 (S3 Table). Moreover, our prior work showed that *tcer-1* mutants’ immunoresistance is not due to reduced pathogen intake [36], supporting a model in which TCER-1 regulates post-infection molecular immunity.

Small RNA-based regulation of antibacterial immunity is an emerging concept across species [14,18,24,69]. Although WAGO Argonautes are not conserved outside of nematodes, some components of this pathway, such as *mut-7*, are conserved from worms to humans [44], raising the possibility that analogous regulatory mechanisms- potentially involving TCER-1 orthologs- link small RNA pathways to antibacterial immunity in other species as well.

## Methods

### *C. elegans* strains and culture

Worm strains were maintained on NGM seeded with *E. coli* strain OP50 using standard methods [70]. RNAi experiments were conducted on NGM supplemented per liter with 1 ml of 1 M IPTG and 1 ml of 100 mg/ml ampicillin and seeded with *E. coli* strain HT115 expressing RNAi constructs or empty vector (pAD12). For survival experiments with FUDR, NGM was supplemented with 100 μg/ml FUDR [36].

### Pathogen stress assays

Pathogen survival assays were performed using the *P. aeruginosa* PA14 slow-killing model [36,71,72]. Kaplan–Meier survival analyses were performed using OASIS 2 [73] with *p* values calculated by the log-rank (Mantel–Cox) test and multiple corrections applied using Bonferroni procedure. Graphs were plotted using GraphPad Prism v9.

### Microscopy and fluorescence quantitation

Day 1 adult TCER-1::GFP worms were immobilized with levamisole (10 mM) and imaged at 20 × magnification on a Leica DMi8 microscope (LAS X software), examining 8–12 animals/condition. Nuclear GFP intensity was quantified using Fiji (ImageJ). Data from two independent biological replicates (≥160 nuclei/strain/replicate) were analyzed in GraphPad Prism 11 using two-way ANOVA.

### RNA seq

sRNA-seq libraries were prepared from size-selected 16–30-nt RNAs isolated from gravid adults by Trizol extraction with chloroform and isopropanol precipitation using the NEBNext Multiplex Small RNA Library Prep Set (NEB, Cat# E7300S) and sequenced on an Illumina NextSeq 500 (Single-End, 75 cycles). mRNA-seq libraries were prepared from rRNA-depleted, DNase-treated RNA using the NEBNext Ultra II Directional RNA Library Prep Kit (NEB, Cat# E7760S) and sequenced on Illumina HiSeq (Paired-End, 150 cycles). sRNA-seq data was processed with the tinyRNA pipeline and mRNA-seq data with a custom RSEM/STAR and DESeq2 pipeline, both using the WormBase WS279 genome release [74–85]. Integrative sRNA/mRNA analysis used the RNA-integrate pipeline (https://github.com/MontgomeryLab/RNA-integrate) [80,81,84]. Full details of library preparation, bioinformatic processing, and integrative analysis are provided in Supplementary Methods in S1 Appendix.

## Supporting information

S1 Fig Survival of *tcer-1(glm27)* (blue) mutants following PA14 infection compared to wildtype animals. Data from two independent trials is summarized in the table showing mean survival in hours (mean) and standard error from the mean (SEM). n = observed/total (see Methods for details). Data from Trial 2 is plotted in the graph. *p* values were calculated using the log-rank method (Mantel Cox).
(TIFF)

S2 Fig Expression and regulation of *scrm-4.* **(A)** mRNA and sRNA read distribution across *scrm-4* and *tcer-1* in wildtype whole adult animals or dissected distal gonads. *mex-5* is shown as a germline-specific reference and *myo-3* as a soma-specific reference. One of three biological replicates is shown. Reads normalized by million mapped reads in each library. **(B)** Ahringer lab genome browser screenshot of ATAC- and CHIP-seq read distribution across the *scrm-4* locus. **(C)** Average log_2_ *scrm-4* 22G-RNA reads in various Argonaute coIPs relative to cell lysates. Reads were normalized by library size. n = 2 biological replicates.
(TIF)

S1 Appendix Supplementary Methods. (DOCX)

S1 Table Impact of *tcer-1* RNAi on survival of *ppw-1* and *rrf-1* mutants on PA14. (DOCX)

S2 Table Impact of *ppw-1* and *rrf-1* mutations on survival of *tcer-1* mutants on PA14. (DOCX)

S3 Table Impact of loss of small RNA biogenesis factors on worm survival on PA14. (DOCX)

S4 Table Differential expression analysis of small RNAs in *tcer-1* mutants vs wildtype. (XLSX)

S5 Table Geometric mean-normalized counts and differential expression analysis of mRNA in *tcer-1* mutants vs wild type. (XLSX)

S6 Table Integrative analysis of 22G-RNA and WAGO target mRNA expression in *tcer-1(tm1452)* vs wildtype. (XLSX)

S7 Table Integrative analysis of 22G-RNA and WAGO target mRNA expression in *tcer-1(glm27)* vs wildtype. (XLSX)

S8 Table Survival of *scrm-4* mutants upon inactivation of *tcer-1* and WAGO 22G-RNA factors. (DOCX)

S9 Table Impact of *wago-1* and *sago-2* depletion on worm survival on PA14. (DOCX)
