# Supplementary figures and images for: A TCER-1-siRNA regulatory axis suppresses antibacterial innate immunity in C. elegans
Source: PLoS Pathog. 2026 Jul 28;22(7):e1013972. doi: 10.1371/journal.ppat.1013972 (PMC13426946; doi:10.1371/journal.ppat.1013972)

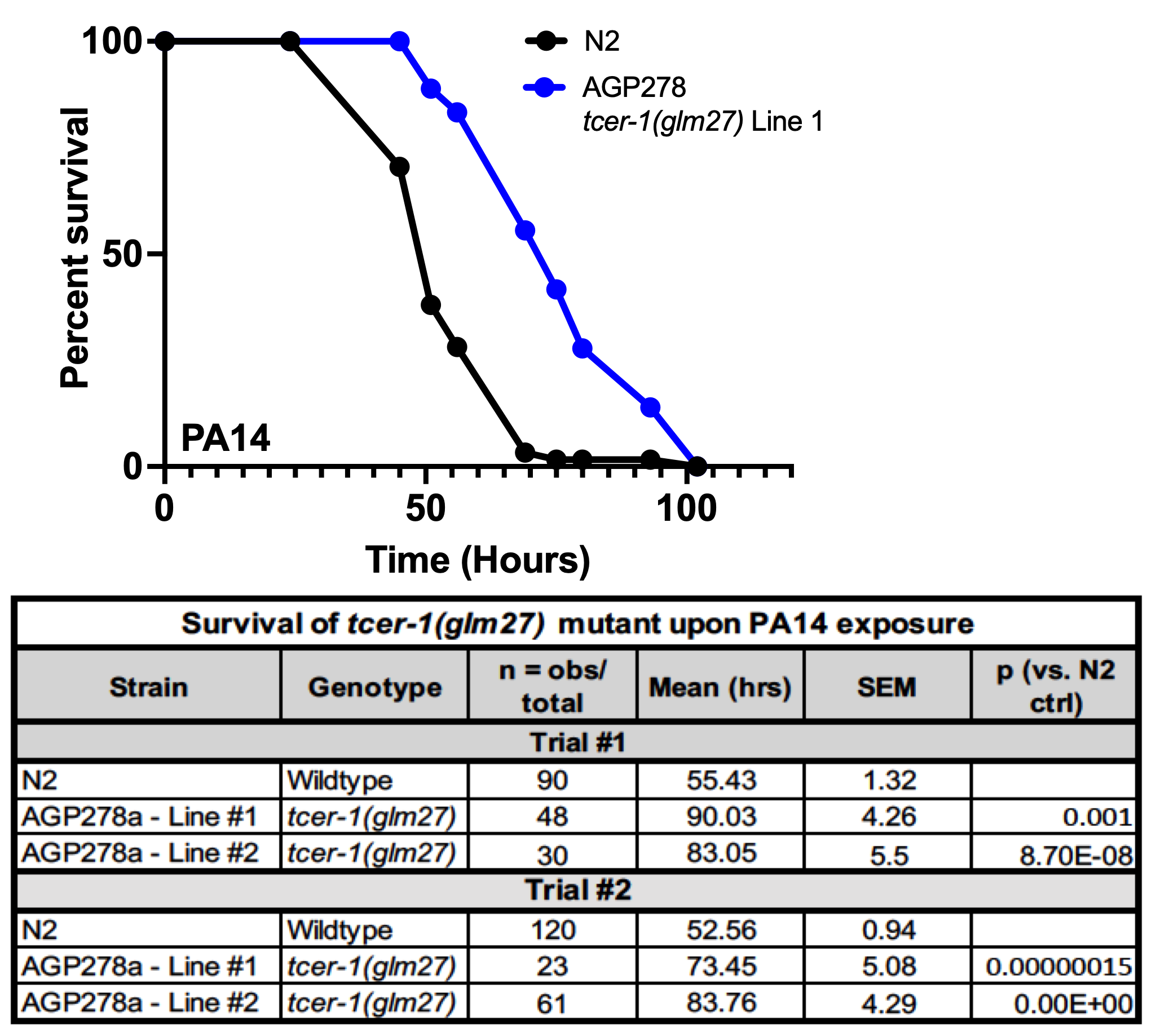

Supplement: S1 Fig — Data from two independent trials is summarized in the table showing mean survival in hours (mean) and standard error from the mean (SEM). n = observed/total (see Methods for details). Data from Trial 2 is plotted in the graph. p values were calculated using the log-rank method (Mantel Cox). (TIFF) [file ppat.1013972.s001.tiff]

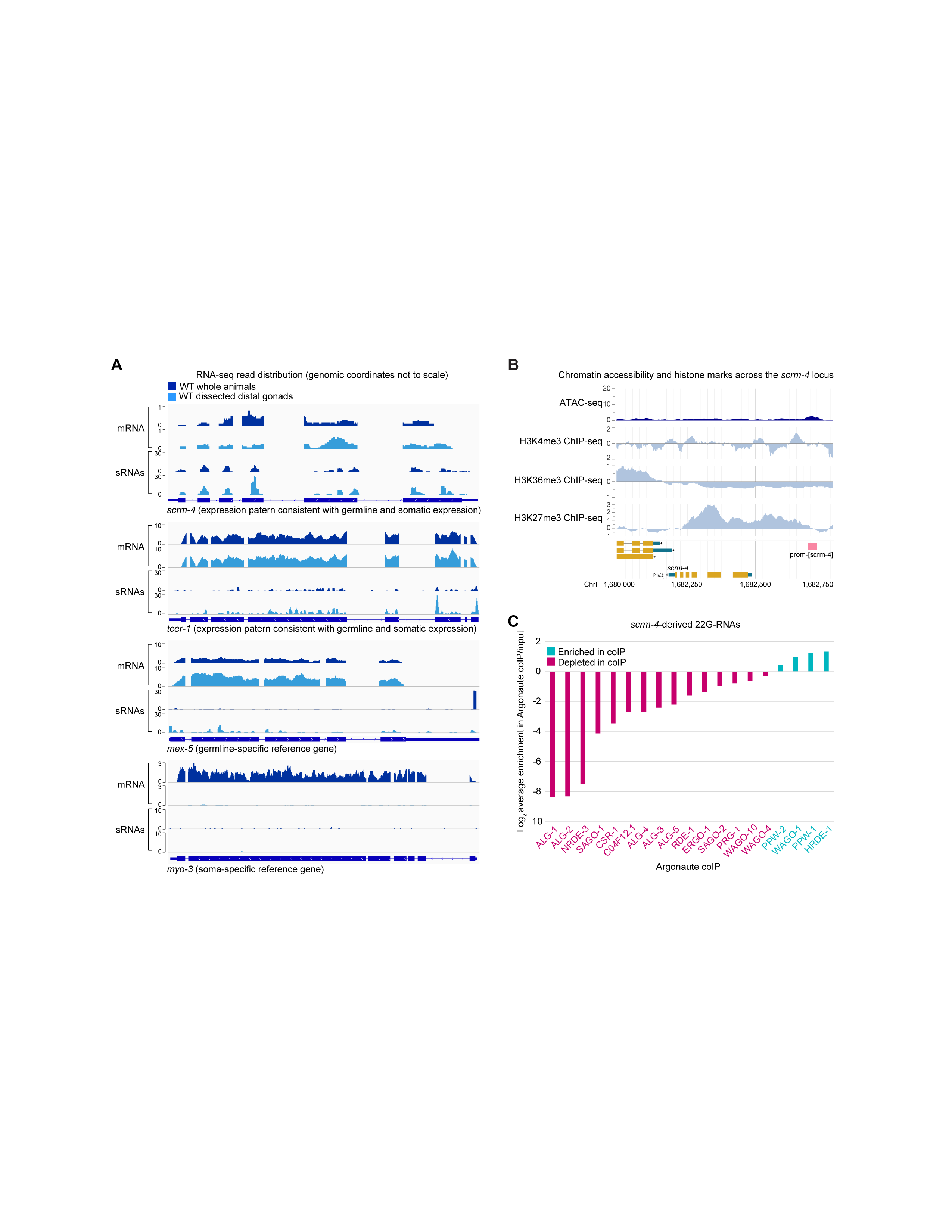

Supplement: S2 Fig — (A) mRNA and sRNA read distribution across scrm-4 and tcer-1 in wildtype whole adult animals or dissected distal gonads. mex-5 is shown as a germline-specific reference and myo-3 as a soma-specific reference. One of three biological replicates is shown. Reads normalized by million mapped reads in each library. (B) Ahringer lab genome browser screenshot of ATAC- and CHIP-seq read distribution across the scrm-4 locus. (C) Average log2 scrm-4 22G-RNA reads in various Argonaute coIPs relative to cell lysates. Reads were normalized by library size. n = 2 biological replicates. (TIF) [file ppat.1013972.s002.tif]
