# Supplementary material for: A TCER-1-siRNA regulatory axis suppresses antibacterial innate immunity in C. elegans
Source: PLoS Pathog. 2026 Jul 28;22(7):e1013972. doi: 10.1371/journal.ppat.1013972 (PMC13426946; doi:10.1371/journal.ppat.1013972)
Supplement: S1 Appendix — (DOCX) [file ppat.1013972.s003.docx]

**APPENDIX**

**A TCER-1-siRNA Regulatory Axis Suppresses Antibacterial Innate Immunity in *C. elegans***

Nikki Naim^1^, Francis R.G. Amrit^1^, Mayur N. Devare^1, $^, Guled A. Osman^1, $^, Laura L. Bahr^1^, Hannah Henry^1^, Brooke E. Montgomery^2^, Spencer M. Kuhn^2^, Taiowa A. Montgomery^2,^ *, and Arjumand Ghazi^1, 3,^ *

^1^Department of Pediatrics, University of Pittsburgh School of Medicine; John G. Rangos Sr. Research Center, One Children's Hospital Drive, 4401 Penn Avenue, Pittsburgh, PA 15224

^2^Department of Biology, Colorado State University, Fort Collins, CO 80523, USA

^3^Department of Cell Biology and Physiology, University of Pittsburgh School of Medicine; 3500 Terrace Street, S362 Biological Science Tower, Pittsburgh, PA 15261

*** Corresponding author:** [**ghazia@pitt.edu**](mailto:ghazia@pitt.edu)

[**Tai.Montgomery@colostate.edu**](mailto:Tai.Montgomery@colostate.edu)

**^$^ Equal contribution**

**SUPPLEMENTARY METHODS:**

**Pathogen stress assays:** Pathogen survival assays were performed using the P. aeruginosa PA14 slow-killing model [1-3]. PA14 was streaked from -80°C stocks onto LB agar, incubated overnight at 37°C, stored at 4°C (≤1 week), and single colonies were grown in King’s Broth (16–18 h, 37°C, shaking). Cultures (20 µL) were seeded onto slow-killing plates (modified NGM, 0.35% peptone), incubated at 37°C for 24 h and then at room temperature for 24 h. Age-matched animals were grown on OP50 or HT115-RNAi plates to L4, designated Day 0 of adulthood, and transferred (≥100 animals; 20–30/plate) to five PA14 plates per strain. For FUDR assays, L4 animals were placed on FUDR plates for 24 h at 15°C prior to transfer. Survival was scored every 6–12 h at 25°C or 20°C, with daily transfers to fresh PA14 plates for the first 3–4 days to separate adults from progeny. Animals that exploded, bagged, crawled off plates, or became contaminated were censored. Kaplan–Meier survival analyses were performed using OASIS 2 [4] with *p* values calculated by the log-rank (Mantel–Cox) test and multiple corrections applied using Bonferroni procedure. Graphs were plotted using GraphPad Prism v9.

***tcer-1(glm27)* Creation.** A *tcer-1* full-gene deletion allele was generated by CRISPR-Cas9 genome editing as described previously [5]. Two guide RNAs were designed to cut at positions flanking the coding region 50 base pair upstream of the start codon (5’: AAAACAGTAGTCTAGCATAA) and 516 base pairs downstream of the stop codon (3’: TCCAACGATAAATTTGAGTA). crRNA and tracrRNA. A repair template (*ttatagagctcaactagatatagttttgaaacttttcgtaattccattatagtacgggtagcacatgaaaacgaaatttccaaaaaacgcttatttctcg*) carrying homology arms flanking the deletion boundaries was co-injected to direct precise removal of the tcer-1 coding sequence via homology-directed repair (HDR). Deletion alleles were identified by PCR using primers flanking the targeted region and confirmed by sequencing to create resulting allele, tcer-1(glm27).

**RNA isolation:** Animals were synchronized by bleach treatment and hatched in M9 until arrested as L1 larvae. Synchronized larvae were plated on NGM plates containing OP50 and grown to gravid adult stage (72 hours post L1 synchronization). Animals were washed three times in M9 buffer, flash frozen in liquid nitrogen, and lysed in Trizol. RNA was isolated using two rounds of chloroform extraction followed by isopropanol precipitation.

**sRNA-seq data analysis:** sRNA sequencing data was processed using the tinyRNA pipeline with default settings and *C. elegans* WS279 genome release [6-11]. 22G-RNA and other small RNA annotations utilized GFF3-formatted annotations available from Knittel et al [12]. 22G-RNAs were defined as antisense-strand 21-23-nt reads containing the 5′-G hallmark of this class of small RNA. Differential expression analysis was done with DESeq2 within the tinyRNA pipeline using the Wald test significance assessment [13]. *p* values were adjusted for multiple testing using the Benjamini-Hochberg procedure. Plotting was done using Matplotlib, R, and IGV [14-16].

**sRNA-seq library preparation:** 16-30-nt RNAs were size selected from total RNA on 17% denaturing polyacrylamide gels. Small RNAs were treated with RNA polyphosphatase to reduce 5′ di- and triphosphates to monophosphates to enable 5′ adapter ligation. Sequencing libraries were prepared with the NEBNext Multiplex Small RNA Library Prep Set for Illumina (NEB, Cat # E7300S). Libraries were size selected on 10% polyacrylamide gels and sequenced on an Illumina NextSeq 500 sequencer (High Output Kit, Single-End, 75 Cycles).

**mRNA-seq library preparation:** rRNA was depleted using the Ribo-Zero rRNA Removal Kit (Human/Mouse/Rat, Illumina Cat # MRZH11124). rRNA-depleted RNA was DNase treated and size selected (>200 nt) to remove 5S rRNA and tRNA using RNA the Clean & Concentrator-5 Kit (Zymo Research, Cat # R1015). RNA-seq libraries were prepared using the NEBNext Ultra II Directional RNA Library Prep Kit for Illumina (NEB, Cat # E7760S). Samples were sequenced on an Illumina HiSeq (Paired-End, 150 Cycles).

**mRNA-seq data analysis:** fastp was used to remove adapters and filter low quality data (fastp -w 16 -q 30 -u 70 -l 30 -r -W 4 -M 20) [8]. Reads were mapped to the *C. elegans* genome (Wormbase release WS279) and transcripts were quantified using RSEM v1.3.1 with STAR v2.7.10b as the aligner [17, 18]. Differential expression analysis was done using DESeq2 v1.50.2 [9, 13].

**Integrative analysis of sRNA and mRNA data:** Integrative analysis of sRNA- and mRNA-seq data was done using the RNA-integrate pipeline (available from <https://github.com/MontgomeryLab/RNA-integrate>). Within the pipeline, differential expression analysis of sRNA-seq and mRNA-seq data were done in parallel with DESeq2 v1.50.2 using the counts tables from the tinyRNA and RSEM analyses described above [11, 13, 18] . Changes in WAGO 22G-RNAs levels were compared to changes in the corresponding mRNAs in *tcer-1* mutants relative to wildtype and data was plotted in Plotly.js v2.11.1 based on fold change, read abundance, and *p* value, as indicated in the plots.

**REFERENCES**

1. Amrit FRG, Naim N, Ratnappan R, Loose J, Mason C, Steenberge L, et al. The Longevity-Promoting Factor, TCER-1, Widely Represses Stress Resistance and Innate Immunity. Nature Communications. 2019;10(1):3042. Epub 2019/07/19. doi: 10.1038/s41467-019-10759-z. PubMed PMID: 31316054; PubMed Central PMCID: PMCPMC6637209.

2. Keith SA, Amrit FR, Ratnappan R, Ghazi A. The C. elegans healthspan and stress-resistance assay toolkit. Methods. 2014;68(3):476-86. Epub 20140413. doi: 10.1016/j.ymeth.2014.04.003. PubMed PMID: 24727065.

3. Tan MW, Ausubel FM. Caenorhabditis elegans: a model genetic host to study Pseudomonas aeruginosa pathogenesis. Curr Opin Microbiol. 2000;3(1):29-34. doi: 10.1016/s1369-5274(99)00047-8. PubMed PMID: 10679415.

4. Han SK, Lee D, Lee H, Kim D, Son HG, Yang JS, et al. OASIS 2: online application for survival analysis 2 with features for the analysis of maximal lifespan and healthspan in aging research. Oncotarget. 2016;7(35):56147-52. doi: 10.18632/oncotarget.11269. PubMed PMID: 27528229; PubMed Central PMCID: PMCPMC5302902.

5. Au V, Li-Leger E, Raymant G, Flibotte S, Chen G, Martin K, et al. CRISPR/Cas9 Methodology for the Generation of Knockout Deletions in Caenorhabditis elegans. G3 Genes|Genomes|Genetics. 2019;9(1):135-44. doi: 10.1534/g3.118.200778.

6. Anders S, Pyl PT, Huber W. HTSeq--a Python framework to work with high-throughput sequencing data. Bioinformatics. 2015;31(2):166-9. Epub 20140925. doi: 10.1093/bioinformatics/btu638. PubMed PMID: 25260700; PubMed Central PMCID: PMCPMC4287950.

7. Chen S, Zhou Y, Chen Y, Gu J. fastp: an ultra-fast all-in-one FASTQ preprocessor. Bioinformatics. 2018;34(17):i884-i90. doi: 10.1093/bioinformatics/bty560. PubMed PMID: 30423086; PubMed Central PMCID: PMCPMC6129281.

8. Langmead B, Trapnell C, Pop M, Salzberg SL. Ultrafast and memory-efficient alignment of short DNA sequences to the human genome. Genome Biol. 2009;10(3):R25. Epub 20090304. doi: 10.1186/gb-2009-10-3-r25. PubMed PMID: 19261174; PubMed Central PMCID: PMCPMC2690996.

9. Lee GY, Ham S, Lee SV. Brief guide to RNA sequencing analysis for nonexperts in bioinformatics. Mol Cells. 2024;47(5):100060. Epub 20240416. doi: 10.1016/j.mocell.2024.100060. PubMed PMID: 38614390; PubMed Central PMCID: PMCPMC11091515.

10. Sternberg PW, Van Auken K, Wang Q, Wright A, Yook K, Zarowiecki M, et al. WormBase 2024: status and transitioning to Alliance infrastructure. Genetics. 2024;227(1). doi: 10.1093/genetics/iyae050. PubMed PMID: 38573366; PubMed Central PMCID: PMCPMC11075546.

11. Tate AJ, Brown KC, Montgomery TA. tiny-count: a counting tool for hierarchical classification and quantification of small RNA-seq reads with single-nucleotide precision. Bioinform Adv. 2023;3(1):vbad065. Epub 20230518. doi: 10.1093/bioadv/vbad065. PubMed PMID: 37288323; PubMed Central PMCID: PMCPMC10243934.

12. Knittel TL, Montgomery BE, Tate AJ, Deihl EW, Nawrocki AS, Hoerndli FJ, et al. A low-abundance class of Dicer-dependent siRNAs produced from a variety of features in C. elegans. Genome Res. 2024;34(12):2203-16. Epub 20241223. doi: 10.1101/gr.279083.124. PubMed PMID: 39622635; PubMed Central PMCID: PMCPMC11694761.

13. Love MI, Huber W, Anders S. Moderated estimation of fold change and dispersion for RNA-seq data with DESeq2. Genome Biol. 2014;15(12):550. doi: 10.1186/s13059-014-0550-8. PubMed PMID: 25516281; PubMed Central PMCID: PMCPMC4302049.

14. Hunter JD. Matplotlib: A 2D Graphics Environment. Computing in Science & Engineering. 2007;9(3):90-5. doi: 10.1109/MCSE.2007.55.

15. Robinson JT, Thorvaldsdóttir H, Winckler W, Guttman M, Lander ES, Getz G, et al. Integrative genomics viewer. Nat Biotechnol. 2011;29(1):24-6. doi: 10.1038/nbt.1754. PubMed PMID: 21221095; PubMed Central PMCID: PMCPMC3346182.

16. Team RC. R: A language and environment for statistical

## computing. R Foundation for Statistical Computing, Vienna, Austria. 2021.

17. Dobin A, Davis CA, Schlesinger F, Drenkow J, Zaleski C, Jha S, et al. STAR: ultrafast universal RNA-seq aligner. Bioinformatics. 2013;29(1):15-21. Epub 20121025. doi: 10.1093/bioinformatics/bts635. PubMed PMID: 23104886; PubMed Central PMCID: PMCPMC3530905.

18. Li B, Dewey CN. RSEM: accurate transcript quantification from RNA-Seq data with or without a reference genome. BMC Bioinformatics. 2011;12:323. Epub 20110804. doi: 10.1186/1471-2105-12-323. PubMed PMID: 21816040; PubMed Central PMCID: PMCPMC3163565.
