# Supplementary material for: A TCER-1-siRNA regulatory axis suppresses antibacterial innate immunity in C. elegans
Source: PLoS Pathog. 2026 Jul 28;22(7):e1013972. doi: 10.1371/journal.ppat.1013972 (PMC13426946; doi:10.1371/journal.ppat.1013972)
Supplement: S1 Table — (DOCX) [file ppat.1013972.s004.docx]

| **S1 Table.** Impact of *tcer-1* RNAi on survival of *ppw-1* and *rrf-1* mutants on PA14.   \| **Genotype** \| **RNAi** \| **n = obs/total** \| **Mean (hrs)** \| **SEM** \| ***p* (vs. Mutant pAD12 Ctrl)** \| ***p* (vs. N2 pAD12 Ctrl)** \| \| --- \| --- \| --- \| --- \| --- \| --- \| --- \| \| **Trial 1** \| \| \| \| \| \| \| \| N2 \| Ctrl \| 87/113 \| 56.61 \| 1.36 \|  \|  \| \| N2 \| *tcer-1* \| 86/114 \| 64.57 \| 1.51 \|  \| 0.0007 \| \| *rrf-1(ok589)* \| Ctrl \| 97/117 \| 75.99 \| 2.25 \|  \| <0.0001 \| \| *rrf-1(ok589)* \| *tcer-1* \| 119/136 \| 83.89 \| 1.75 \| 0.3887 \|  \| \| *ppw-1(pk1425)* \| Ctrl \| 118/126 \| 76.33 \| 1.77 \|  \| <0.0001 \| \| *ppw-1(pk1425)* \| *tcer-1* \| 132/148 \| 70 \| 1.32 \| 0.0197 \|  \| \| **Trial 2** \| \| \| \| \| \| \| \| N2 \| Ctrl \| 110/134 \| 60.93 \| 0.95 \|  \|  \| \| N2 \| *tcer-1* \| 129/157 \| 67.91 \| 1.09 \| 0.00004 \|  \| \| *rrf-1(ok589)* \| Ctrl \| 114/136 \| 77.45 \| 1.67 \| <0.0001 \|  \| \| *rrf-1(ok589)* \| *tcer-1* \| 117/154 \| 77.3 \| 1.33 \|  \| 1 \| \| *ppw-1(pk1425)* \| Ctrl \| 123/144 \| 72.14 \| 1.46 \| <0.0001 \|  \| \| *ppw-1(pk1425)* \| *tcer-1* \| 1412/154 \| 72.46 \| 1.2 \|  \| 1 \| \| **Trial 3** \| \| \| \| \| \| \| \| N2 \| Ctrl \| 63/81 \| 58.52 \| 1.48 \|  \|  \| \| N2 \| *tcer-1* \| 78/109 \| 69.23 \| 1.48 \|  \| 0.0000068 \| \| *rrf-1(ok589)* \| Ctrl \| 93/108 \| 83.39 \| 2.16 \|  \| <0.0001 \| \| *rrf-1(ok589)* \| *tcer-1* \| 104/131 \| 88.44 \| 2.12 \| 0.3968 \|  \| \| *ppw-1(pk1425)* \| Ctrl \| 99/112 \| 71.2 \| 1.95 \|  \| 0.000007 \| \| *ppw-1(pk1425)* \| *tcer-1* \| 110/121 \| 70.14 \| 1.62 \| 1 \|  \| \| **Trial 4** \| \| \| \| \| \| \| \| N2 \| Ctrl \| 131/167 \| 59.33 \| 1.16 \|  \|  \| \| N2 \| *tcer-1* \| 143/161 \| 76.23 \| 1.52 \|  \| <0.0001 \| \| *rrf-1(ok589)* \| Ctrl \| 137/170 \| 79.77 \| 1.63 \|  \| <0.0001 \| \| *rrf-1(ok589)* \| *tcer-1* \| 144/164 \| 82.62 \| 1.69 \| 0.807 \|  \| \| *ppw-1(pk1425)* \| Ctrl \| 144/157 \| 84.43 \| 1.74 \|  \| <0.0001 \| \| *ppw-1(pk1425)* \| *tcer-1* \| 134/151 \| 87.16 \| 1.99 \| 1 \|  \| \| **Trial 5** \| \| \| \| \| \| \| \| N2 \| Ctrl \| 75/151 \| 57.93 \| 1.09 \|  \|  \| \| N2 \| *tcer-1* \| 73/175 \| 77.06 \| 1.8 \|  \| 0.0001 \| \| *rrf-1(ok589)* \| Ctrl \| 97/155 \| 81.81 \| 2.1 \|  \| 0.0001 \| \| *rrf-1(ok589)* \| *tcer-1* \| 123/160 \| 82.11 \| 1.24 \| 0.2123 \|  \| \| *ppw-1(pk1425)* \| Ctrl \| 71/140 \| 70.34 \| 2.11 \|  \| 0.000002 \| \| *ppw-1(pk1425)* \| *tcer-1* \| 111/155 \| 78.32 \| 1.32 \| 0.0019 \|  \| \| **Trial 6** \| \| \| \| \| \| \| \| N2 \| Ctrl \| 99/112 \| 64.21 \| 1.73 \|  \|  \| \| N2 \| *tcer-1* \| 92/97 \| 78.73 \| 1.39 \|  \| <0.0001 \| \| *rrf-1(ok589)* \| Ctrl \| 100/115 \| 78.17 \| 2.8 \|  \| 0.0001 \| \| *rrf-1(ok589)* \| *tcer-1* \| 126/140 \| 93.07 \| 2.4 \| 0.0076 \|  \| \| *ppw-1(pk1425)* \| Ctrl \| 82/123 \| 86.34 \| 2.54 \|  \| <0.0001 \| \| *ppw-1(pk1425)* \| *tcer-1* \| 149/180 \| 91.81 \| 1.83 \| 0.6764 \|  \| |
| --- | --- | --- | --- | --- | --- | --- | --- | --- | --- | --- | --- | --- | --- | --- | --- | --- | --- | --- | --- | --- | --- | --- | --- | --- | --- | --- | --- | --- | --- | --- | --- | --- | --- | --- | --- | --- | --- | --- | --- | --- | --- | --- | --- | --- | --- | --- | --- | --- | --- | --- | --- | --- | --- | --- | --- | --- | --- | --- | --- | --- | --- | --- | --- | --- | --- | --- | --- | --- | --- | --- | --- | --- | --- | --- | --- | --- | --- | --- | --- | --- | --- | --- | --- | --- | --- | --- | --- | --- | --- | --- | --- | --- | --- | --- | --- | --- | --- | --- | --- | --- | --- | --- | --- | --- | --- | --- | --- | --- | --- | --- | --- | --- | --- | --- | --- | --- | --- | --- | --- | --- | --- | --- | --- | --- | --- | --- | --- | --- | --- | --- | --- | --- | --- | --- | --- | --- | --- | --- | --- | --- | --- | --- | --- | --- | --- | --- | --- | --- | --- | --- | --- | --- | --- | --- | --- | --- | --- | --- | --- | --- | --- | --- | --- | --- | --- | --- | --- | --- | --- | --- | --- | --- | --- | --- | --- | --- | --- | --- | --- | --- | --- | --- | --- | --- | --- | --- | --- | --- | --- | --- | --- | --- | --- | --- | --- | --- | --- | --- | --- | --- | --- | --- | --- | --- | --- | --- | --- | --- | --- | --- | --- | --- | --- | --- | --- | --- | --- | --- | --- | --- | --- | --- | --- | --- | --- | --- | --- | --- | --- | --- | --- | --- | --- | --- | --- | --- | --- | --- | --- | --- | --- | --- | --- | --- | --- | --- | --- | --- | --- | --- | --- | --- | --- | --- | --- | --- | --- | --- | --- | --- | --- | --- | --- | --- | --- | --- | --- | --- | --- | --- | --- | --- | --- | --- | --- | --- | --- | --- | --- | --- | --- | --- | --- | --- | --- | --- | --- | --- | --- | --- | --- | --- | --- | --- | --- | --- | --- | --- | --- | --- | --- |
