# Supplementary material for: A TCER-1-siRNA regulatory axis suppresses antibacterial innate immunity in C. elegans
Source: PLoS Pathog. 2026 Jul 28;22(7):e1013972. doi: 10.1371/journal.ppat.1013972 (PMC13426946; doi:10.1371/journal.ppat.1013972)
Supplement: S2 Table — (DOCX) [file ppat.1013972.s005.docx]

| **S2 Table.** Impact of loss of *tcer-1* on survival of *ppw-1* and *rrf-1* mutants on PA14.   \| **Strain** \| **Genotype** \| **n = obs/total** \| **Mean (hrs)** \| **SEM** \| **p (vs N2)** \| ***p***  **(vs *tcer-1*)** \| \| --- \| --- \| --- \| --- \| --- \| --- \| --- \| \| **Trial 1** \| \| \| \| \| \| \| \| N2 \| Wildtype \| 108/130 \| 74.9 \| 1.34 \|  \|  \| \| CF1266 \| *tcer-1(tm1452)* \| 91/120 \| 103.91 \| 2.45 \| <0.0001 \|  \| \| NL3511 \| *ppw-1(pk1425)* \| 119/146 \| 97.77 \| 1.96 \| <0.0001 \|  \| \| AGP274 \| *ppw-1; tcer-1* \| 99/123 \| 112.97 \| 2.94 \| <0.0001 \| 0.05 \| \| RB798 \| *rrf-1(ok589)* \| 113/137 \| 97.99 \| 1.89 \| <0.0001 \|  \| \| AGP273 \| *rrf-1;tcer-1* \| 88/106 \| 108.5 \| 2.55 \| <0.0001 \| 0.63 \| \| T**rial 2** \| \| \| \| \| \| \| \| N2 \| Wildtype \| 116/143 \| 88.97 \| 2.01 \|  \|  \| \| CF1266 \| *tcer-1(tm1452)* \| 96/111 \| 102.76 \| 2.3 \| 0.0002 \|  \| \| NL3511 \| *ppw-1(pk1425)* \| 134/148 \| 102.95 \| 2.38 \| 0.0001 \|  \| \| AGP274 \| *ppw-1; tcer-1* \| 95/116 \| 106.68 \| 2.94 \| <0.0001 \| 0.97 \| \| RB798 \| *rrf-1(ok589)* \| 120/140 \| 118.33 \| 2.88 \| <0.0001 \|  \| \| AGP273 \| *rrf-1; tcer-1* \| 127/148 \| 102.84 \| 2.38 \| 0.0001 \| 1 \| \| **Trial 3** \| \| \| \| \| \| \| \| N2 \| Wildtype \| 149/181 \| 90.81 \| 1.65 \|  \|  \| \| CF1266 \| *tcer-1(tm1452)* \| 113/163 \| 97.57 \| 1.74 \| 0.0354 \|  \| \| NL3511 \| *ppw-1(pk1425)* \| 141/180 \| 86.38 \| 1.44 \| 0.0624 \|  \| \| AGP274 \| *ppw-1; tcer-1* \| 127/181 \| 93.08 \| 1.75 \| 1 \| 0.479 \| \| RB798 \| *rrf-1(ok589)* \| 134/180 \| 96.04 \| 1.51 \| 0.3229 \|  \| \| AGP273 \| *rrf-1;tcer-1* \| 103/154 \| 99.44 \| 1.76 \| 0.007 \| 0.3859 \| |
| --- | --- | --- | --- | --- | --- | --- | --- | --- | --- | --- | --- | --- | --- | --- | --- | --- | --- | --- | --- | --- | --- | --- | --- | --- | --- | --- | --- | --- | --- | --- | --- | --- | --- | --- | --- | --- | --- | --- | --- | --- | --- | --- | --- | --- | --- | --- | --- | --- | --- | --- | --- | --- | --- | --- | --- | --- | --- | --- | --- | --- | --- | --- | --- | --- | --- | --- | --- | --- | --- | --- | --- | --- | --- | --- | --- | --- | --- | --- | --- | --- | --- | --- | --- | --- | --- | --- | --- | --- | --- | --- | --- | --- | --- | --- | --- | --- | --- | --- | --- | --- | --- | --- | --- | --- | --- | --- | --- | --- | --- | --- | --- | --- | --- | --- | --- | --- | --- | --- | --- | --- | --- | --- | --- | --- | --- | --- | --- | --- | --- | --- | --- | --- | --- | --- | --- | --- | --- | --- | --- | --- | --- | --- | --- | --- | --- | --- | --- | --- | --- | --- | --- | --- | --- | --- |
