# Supplementary material for: A TCER-1-siRNA regulatory axis suppresses antibacterial innate immunity in C. elegans
Source: PLoS Pathog. 2026 Jul 28;22(7):e1013972. doi: 10.1371/journal.ppat.1013972 (PMC13426946; doi:10.1371/journal.ppat.1013972)
Supplement: S3 Table — (DOCX) [file ppat.1013972.s006.docx]

| **S3 Table.** Impact of loss of small RNA biogenesis factors on worm survival on PA14.   \| **Strain** \| **Genotype** \| **n = obs/total** \| **Mean (hrs)** \| **SEM** \| **Bonferroni**  ***p* (vs. N2)** \| \| --- \| --- \| --- \| --- \| --- \| --- \| \| **Trial 1** \| \| \| \| \| \| \| N2 \| Wildtype \| 137/155 \| 118.27 \| 2.7 \|  \| \| CF2166 \| *tcer-1(tm1452)* \| 119/170 \| 183.11 \| 4.35 \| <0.001 \| \| NL1810 \| *mut-16(pk710)* \| 127/145 \| 135.95 \| 2.95 \| <0.001 \| \| GR1946 \| *mut-14(pk738) smut-1(tm1301)* \| 139/159 \| 169.7 \| 4.52 \| <0.001 \| \| NL1820 \| *mut-7(pk720)* \| 129/149 \| 138.18 \| 4.44 \| <0.001 \| \| **Trial 2** \| \| \| \| \| \| \| N2 \| Wildtype \| 112/162 \| 111.47 \| 1.92 \|  \| \| CF2166 \| *tcer-1(tm1452)* \| 120/201 \| 151.35 \| 4.2 \| <0.001 \| \| NL1810 \| *mut-16(pk710)* \| 110/141 \| 116.67 \| 3.14 \| 0.6114 \| \| GR1946 \| *mut-14(pk738) smut-1(tm1301)* \| 134/160 \| 141.88 \| 3.7 \| <0.001 \| \| NL1820 \| *mut-7(pk720)* \| 89/115 \| 160.94 \| 5.52 \| <0.001 \| \| WM30 \| *rde-3(ne298)* \| 124/153 \| 101.24 \| 1.64 \| <0.001 \| \| **Trial 3** \| \| \| \| \| \| \| N2 \| Wildtype \| 117/158 \| 137.64 \| 3.34 \|  \| \| CF2166 \| *tcer-1(tm1452)* \| 94/167 \| 166.53 \| 4.48 \| <0.001 \| \| NL1810 \| *mut-16(pk710)* \| 80/122 \| 134.79 \| 3.51 \| 1 \| \| DCL565 \| *rde-1(mkc36)* \| 115/164 \| 127.83 \| 3.04 \| 0.1937 \| \| WM45 \| *rde-1(ne300)* \| 105/134 \| 102.26 \| 1.27 \| <0.001 \| \| WM27 \| *rde-1(ne219)* \| 105/135 \| 115.25 \| 2.01 \| <0.001 \| \| **Trial 4*** \| \| \| \| \| \| \| N2 \| Wildtype \| 99/138 \| 58.56 \| 1.53 \| <0.001 \| \| CF2166 \| *tcer-1(tm1452)* \| 52/136 \| 77.52 \| 2.93 \| <0.001 \| \| NL1810 \| *mut-16(pk710)* \| 121/135 \| 64.21 \| 1.39 \| 0.0309 \| \| GR1946 \| *mut-14(pk738) smut-1(tm1301)* \| 115/138 \| 59.46 \| 1.66 \| 1 \| \| **Trial 5** \| \| \| \| \| \| \| N2 \| Wildtype \| 108/130 \| 74.9 \| 1.34 \|  \| \| SX2499 \| *prde-1(mj207)* \| 71/104 \| 75.84 \| 2 \| 1 \| \| CF2166 \| *tcer-1 (tm1452)* \| 91/120 \| 103.91 \| 2.45 \| <0.001 \| |
| --- | --- | --- | --- | --- | --- | --- | --- | --- | --- | --- | --- | --- | --- | --- | --- | --- | --- | --- | --- | --- | --- | --- | --- | --- | --- | --- | --- | --- | --- | --- | --- | --- | --- | --- | --- | --- | --- | --- | --- | --- | --- | --- | --- | --- | --- | --- | --- | --- | --- | --- | --- | --- | --- | --- | --- | --- | --- | --- | --- | --- | --- | --- | --- | --- | --- | --- | --- | --- | --- | --- | --- | --- | --- | --- | --- | --- | --- | --- | --- | --- | --- | --- | --- | --- | --- | --- | --- | --- | --- | --- | --- | --- | --- | --- | --- | --- | --- | --- | --- | --- | --- | --- | --- | --- | --- | --- | --- | --- | --- | --- | --- | --- | --- | --- | --- | --- | --- | --- | --- | --- | --- | --- | --- | --- | --- | --- | --- | --- | --- | --- | --- | --- | --- | --- | --- | --- | --- | --- | --- | --- | --- | --- | --- | --- | --- | --- | --- | --- | --- | --- | --- | --- | --- | --- | --- | --- | --- | --- | --- | --- | --- | --- | --- | --- | --- | --- | --- | --- | --- | --- | --- | --- | --- | --- | --- | --- | --- | --- | --- | --- |

* PA14 exposure at 20˚C except trial 4 which was done at 25˚C.
