# Supplementary material for: A TCER-1-siRNA regulatory axis suppresses antibacterial innate immunity in C. elegans
Source: PLoS Pathog. 2026 Jul 28;22(7):e1013972. doi: 10.1371/journal.ppat.1013972 (PMC13426946; doi:10.1371/journal.ppat.1013972)
Supplement: S8 Table — (DOCX) [file ppat.1013972.s011.docx]

**S8 Table.** Survival of *scrm-4* mutants upon inactivation of *tcer-1* and WAGO 22G-RNA factors.

| **Genotype** | **RNAi** | **n = obs/total** | **Mean (hrs)** | **SEM** | ***p* (vs. Ctrl EV)** |
| --- | --- | --- | --- | --- | --- |
| **Trial 1*** | | | | | |
| N2 | Ctrl EV | 58/90 | 58.09 | 1.52 |  |
| N2 | *ppw-1* | 52/90 | 69.25 | 1.3 | 0.0000220 |
| N2 | *tcer-1* | 54/90 | 71.21 | 1.95 | 0.0000190 |
| N2 | *mut-16* | 68/90 | 65.59 | 2.22 | 0.3331000 |
| *scrm-4* | Ctrl EV | 52/90 | 67.37 | 2 |  |
| *scrm-4* | *ppw-1* | 41/90 | 71.83 | 2.51 | 0.2959000 |
| *scrm-4* | *tcer-1* | 41/90 | 71.53 | 1.74 | 0.1677000 |
| *scrm-4* | *mut-16* | 41/90 | 72.19 | 2.3 | 0.1424000 |
| **Trial 2** | | | | | |
| N2 | Ctrl EV | 61/89 | 64.75 | 1.7 |  |
| N2 | *ppw-1* | 30/80 | 81.8 | 2.01 | <0.00001 |
| N2 | *tcer-1* | 39/90 | 80.82 | 2.11 | <0.00001 |
| N2 | *mut-16* | 30/93 | 78.75 | 2.2 | <0.00001 |
| *scrm-4* | Ctrl EV | 52/74 | 61.78 | 1.43 |  |
| *scrm-4* | *ppw-1* | 64/81 | 60.03 | 1.48 | 1.0000000 |
| *scrm-4* | *tcer-1* | 56/82 | 60.3 | 1.7 | 1.0000000 |
| *scrm-4* | *mut-16* | 45/82 | 63.91 | 1.42 | 0.9135000 |
| **Trial 3*** | | | | | |
| N2 | Ctrl EV | 80/89 | 59.35 | 1.22 |  |
| N2 | *tcer-1* | 78/90 | 63.94 | 1.83 | 0.0566000 |
| N2 | *wago-1* | 67/81 | 63.41 | 1.81 | 0.1445000 |
| N2 | *sago-2* | 80/89 | 59.29 | 1.27 | 1.0000000 |
| *scrm-4* | Ctrl EV | 88/88 | 53.98 | 0.7 |  |
| *scrm-4* | *tcer-1* | 88/88 | 56.9 | 0.88 | 0.0187000 |
| *scrm-4* | *wago-1* | 83/83 | 58.59 | 0.85 | 0.0003000 |
| *scrm-4* | *sago-2* | 83/88 | 59.46 | 1.09 | 0.0001000 |
| **Trial 4** | | | | | |
| N2 | Ctrl EV | 78/90 | 59.21 | 1.45 |  |
| N2 | *tcer-1* | 71/90 | 61.31 | 1.83 | 1.0000000 |
| N2 | *wago-1* | 70/90 | 58.38 | 1.39 | 1.0000000 |
| N2 | *sago-2* | 76/90 | 58.44 | 1.57 | 1.0000000 |
| *scrm-4* | Ctrl EV | 70/90 | 57.52 | 1.52 |  |
| *scrm-4* | *tcer-1* | 63/90 | 61.81 | 1.4 | 0.2317000 |
| *scrm-4* | *wago-1* | 69/90 | 59.1 | 1.4 | 1.0000000 |
| *scrm-4* | *sago-2* | 66/90 | 67.05 | 1.76 | 0.0036000 |

**FUDR not used*
