# Supplementary material for: A TCER-1-siRNA regulatory axis suppresses antibacterial innate immunity in C. elegans
Source: PLoS Pathog. 2026 Jul 28;22(7):e1013972. doi: 10.1371/journal.ppat.1013972 (PMC13426946; doi:10.1371/journal.ppat.1013972)
Supplement: S9 Table — (DOCX) [file ppat.1013972.s012.docx]

**S9 Table.** Impact of *wago-1* and *sago-2* depletion on worm survival on PA14.

| **Genotype** | **RNAi** | **n = obs/total** | **Mean (hrs)** | **SEM** | **Bonferroni *p* (vs. N2 Ctrl EV)** | **Bonferroni *p* (vs. Ctrl EV)** |
| --- | --- | --- | --- | --- | --- | --- |
| **Trial 1*** | | | | | | |
| N2 | Ctrl EV | 81/90 | 70.67 | 1.09 |  |  |
| N2 | *tcer-1* | 72/90 | 71.02 | 1.98 |  | 1 |
| *wago-1(ok1074)* | Ctrl EV | 79/90 | 94.5 | 2.46 | <0.0001 |  |
| *wago-1(ok1074)* | *tcer-1* | 73/90 | 88.28 | 1.94 |  | 0.04 |
| *sago-2(tor135)* | Ctrl EV | 78/90 | 80.73 | 1.43 | <0.0001 |  |
| *sago-2(tor135)* | *tcer-1* | 73/90 | 79.16 | 1.87 |  | 1 |
| *sago-2(tm894)* | Ctrl EV | 76/90 | 79.79 | 1.11 | <0.0001 |  |
| *sago-2(tm894)* | *tcer-1* | 71/90 | 77.36 | 1.03 |  | 0.56 |
| **Trial 2** | | | | | | |
| N2 | Ctrl EV | 78/90 | 72.62 | 1.41 |  |  |
| N2 | *tcer-1* | 77/90 | 78.61 | 1.28 |  | 0.04 |
| *wago-1(ok1074)* | Ctrl EV | 73/90 | 77.6 | 1.84 | 0.24 |  |
| *wago-1(ok1074)* | *tcer-1* | 82/90 | 83.37 | 1.95 |  | 0.28 |
| *sago-2(tor135)* | Ctrl EV | 79/90 | 72.32 | 1.08 | 0.85 |  |
| *sago-2(tor135)* | *tcer-1* | 72/90 | 75.2 | 1.06 |  | 0.45 |
| *sago-2(tm894)* | Ctrl EV | 68/90 | 75.1 | 1.33 | 0.85 |  |
| *sago-2(tm894)* | *tcer-1* | 75/90 | 78.05 | 1.37 |  | 1 |

| **Trial 1*** | | | | | |
| --- | --- | --- | --- | --- | --- |
| **Genotype** | **RNAi** | **n = obs/total** | **Mean (hrs)** | **SEM** | **Bonferroni *p* (vs. N2 Ctrl EV)** |
| N2 | Ctrl EV | 78/90 | 59.21 | 1.45 |  |
| N2 | *tcer-1* | 71/90 | 61.31 | 1.83 | 1 |
| N2 | *wago-1* | 70/90 | 58.38 | 1.39 | 1 |
| N2 | *sago-2* | 76/90 | 58.44 | 1.57 | 1 |
| **Trial 2** | | | | | |
| N2 | Ctrl EV | 80/89 | 59.35 | 1.22 |  |
| N2 | *tcer-1* | 78/90 | 63.94 | 1.83 | 0.05 |
| N2 | *wago-1* | 67/81 | 63.41 | 1.81 | 0.14 |
| N2 | *sago-2* | 80/89 | 59.29 | 1.27 | 1 |

**FUDR not used*
