# Supplementary material for: A TCER-1-siRNA regulatory axis suppresses antibacterial innate immunity in C. elegans
Source: PLoS Pathog. 2026 Jul 28;22(7):e1013972. doi: 10.1371/journal.ppat.1013972 (PMC13426946; doi:10.1371/journal.ppat.1013972)
Supplement: Attachment — Submitted filename: RESP_REVIEWERS_23JUN26_NaimEtal_FINAL.docx [file ppat.1013972.s014.docx]

# Naim et al (*PPATHOGENS-D-26-00272*) RESPONSE TO REVIEWERS

We are sincerely grateful to the reviewers for their insightful and constructive feedback which has substantially strengthened the clarity and thoroughness of our manuscript. Below we provide detailed, point-by-point responses addressing each of their comments (in blue).

# Part I - Summary

**Reviewer #1:** In this manuscript entitled “A TCER-1-siRNA Regulatory Axis Suppresses Antibacterial Innate Immunity in C. elegans,” the authors investigated how endogenous siRNA pathways modulate antibacterial immunity against Pseudomonas aeruginosa PA14. The authors identified endogenous small RNAs, WAGO Argonaute-associated 22G-RNAs, that repress antibacterial immunity. The authors found that multiple components required for WAGO 22G-RNA biogenesis suppress host resistance through TCER-1, which is transcription elongation and splicing factor. They also showed that TCER-1 targets a restricted set of immune-relevant effectors, such as scrm-4, rather than inducing widespread gene silencing. The authors highlighted a TCER-1-siRNA regulatory axis in which TCER-1 promotes WAGO 22G-RNA biogenesis, thereby repressing antibacterial immunity.

**Reviewer #2:** In this manuscript, Naim and colleagues provide new insights into the role of small interfering RNAs (siRNAs) in C. elegans antibacterial defense. They demonstrate that TCER-1, the C. elegans homolog of human transcription elongation and splicing factor TCERG1, suppresses host defense against Pseudomonas aeruginosa PA14 via the WAGO 22G-RNA pathway. The role of TCER-1 as a repressor of host defense against bacterial pathogens had been established in previous work by the same group. High-throughput small RNA and mRNA sequencing data from two tcer-1 mutants revealed that expression of WAGO 22G-RNA pathway genes was not down-regulated in tcer-1 mutants. Thus, TCER-1 probably does not directly affect the WAGO 22G-RNA pathway, but rather indirectly influences stability of 22G-RNAs or the processing of WAGO targets. The exact mechanism of the interaction remains unclear. However, the authors also find that the WAGO target scrm-4, which encodes a phospholipid translocase, contributes to pathogen defenses in a TCER-1-dependent manner.

These results are significant and novel. While the role of miRNAs in antibacterial host defense in Caenorhabditis elegans is well established, endogenous siRNAs have been primarily studied in the context of antiviral defense. Although emerging evidence suggests that endogenous siRNA pathways may influence antibacterial immunity, their precise contribution to antibacterial host defense remains unclear. This study provides direct evidence of endogenous siRNA participation in antibacterial pathways, via interaction with TCER-1 and the WAGO target gene scrm-4. It significantly broadens our understanding of the role of endo-siRNAs in host-pathogen interactions and is thus of high importance and broad interest to the community in this field. The manuscript is clearly written, and the experiments have been performed rigorously and are presented in a convincing and comprehensive manner. I only have two comments, including a suggestion for an additional experiment. Other than that, publication of the presented results as they stand is in my opinion justified.

**Reviewer #3:** In this manuscript the authors link the 22GRNA pathway in C. elegans to pathogen resistance, via the factor TCER-1 which acts upstream of 22G pathways. They provide convincing evidence for the alteration of 22G-RNAs against a small number of genes when TCER-1 is inactive and, nicely, are able to trace the effect on pathogen resistance to a particular target, scrm-4. The genetics experiments, statistical and computational analyses are excellent and the paper is written clearly and logically, without overstating the results

# Part II – Major Points

**Reviewer #1**

1. In Figure 1E, the authors conclude that TCER-1::GFP expression is unchanged by ppw-1 or rrf-1 mutations, but the evidence is presented mainly as representative images. I recommend that the authors include a quantitative analysis of GFP expression from the TCER-1::GFP translation reporter. This would strengthen the conclusion that ppw-1 or rrf-1 mutations do not alter TCER-1 expression.

We thank the reviewer for this valuable suggestion, and have quantified TCER-1::GFP expression in the *ppw-1* and *rrf-1* mutants. The results revealed that TCER-1::GFP levels showed a very modest reduction in the intestinal nuclei of *rrf-1* as well as *ppw-1* mutants. This data, along with the observation that *rrf-1* mRNA levels are modestly reduced in *tcer-1* mutants in our RNAseq studies, suggests there could be a mild regulatory relationship between these genes. The data has been added as Fig. 1F (below) and the Results section revised accordingly (Pg. 6; Lines 94-101).

2. Fourteen targets show strong anti-correlation between 22G-RNAs and mRNA levels, and three, including scrm-4, exhibit changes greater than four-fold. It remains unclear why scrm-4 was prioritized over the others. Please write the justification for that. The epistasis data indicate that scrm-4 is required for tcer-1 RNAi-mediated immunoresistance. Can they add similar experimental results using WAGO mutants? Overexpression study will also strengthen the role of scrm-4 as a downstream target. If the authors do not have such genetric tools for testing these, it will be nice to add discussion points regarding this issue.

Justification for *scrm-4* selection: As mentioned by the reviewer, of the 14 genes that showed anti-correlation in 22G-RNA and mRNA expression levels in both *tcer-1* mutants, only three genes - F15D4.5, Y102A5C.36, and scrm-4 - displayed >4-fold reductions in associated 22G-RNAs accompanied by >4-fold increases in mRNA abundance. We therefore prioritized these genes as the most likely functionally relevant downstream effectors. However, whereas F15D4.5 and Y102A5C.36 encode poorly characterized proteins lacking conserved domains or obvious functional annotations, scrm-4 encodes a phospholipid scramblase orthologous to human PLSCR3, a protein implicated in membrane phospholipid translocation, mitochondrial structure and function, and apoptosis. The combination of strong genetic regulation, evolutionary conservation, and a defined molecular function made scrm-4 a particularly compelling candidate for further study. Moreover, lipid metabolism is a well-established regulator of C. elegans immunity, and our recent work demonstrated that TCER-1 promotes phospholipid remodeling to shape immunometabolic responses (*Bahr et al., PLoS. Gen. 2025; PMID: 41385585*). Together, the (i) strong regulatory signature, (ii) functional annotation and evolutionary conservation, and (iii) direct biological relevance to TCER-1-mediated immune regulation led us to focus on *scrm-4*. We have updated the manuscript to explain this rationale more clearly (Pg. 10, Lines 171-187).

*scrm-4* epistasis with WAGO mutants and overexpression: We assessed the epistasis relationship of *scrm-4* with *ppw-1* and *mut-16*. While RNAi inactivation of both genes enhanced survival of WT animals following PA14 infection, *scrm-4* mutants did not exhibit a significant increase in either case, suggesting that *scrm-4* is required broadly for the enhanced PA14 resistance induced by 22G-RNA pathway downregulation. This data is shown below and included in the revised manuscript as Fig. 4G,H, Table S8 (Pg. 10, Lines 189-191). We were unable to create *scrm-4* overexpression strains in time for a timely resubmission.

**Fig. 4G, H. Left:** Survival of WT animals on Ctrl RNAi (black, m = 64.75 ± 1.7, n = 61/89), *tcer-1* RNAi (blue, m = 80.82 ± 2.11, n = 39/90), *ppw-1* RNAi (red, m = 81.8 ± 2.01, n = 30/80), *mut-16* RNAi (plum, m = 78.85 ± 2.2, n = 30/93). **Right:** Survival of *scrm-4* mutants on Ctrl RNAi (black, m = 61.78 ± 1.43, n = 52/74), *tcer-1* RNAi (blue, m = 60.3 ± 1.7, n= 56/82), *ppw-1* RNAi (red, m = 60.03 ± 1.48, n= 64/81), *mut-16* RNAi (plum, m = 63.9 ± 1.42, n= 45/82). *** P <0.0001. ns: not significant.

**Reviewer #2**

The study focuses on WAGO Argonaute-associated siRNAs and is based on the intriguing observation that the ppw-1 mutant of the WAGO 22G-RNA pathway, which is used in C. elegans for germline- vs. soma-specific gene-knockdown by RNAi, exhibited clearly decreased susceptibility to infection with P. aeruginosa PA14. PPW-1 is a member of the WAGO clade in C. elegans. Of the 13 AGOs in the WAGO clade, SAGO-2 and PPW-1 were previously shown to be involved in defense against PA14 infection (both sago-2 and ppw-1 mutants are resistant to PA14 infection; Seroussi et al., 2023 [https://doi.org/10.7554/eLife.83853).](https://doi.org/10.7554/eLife.83853)) Therefore, it would be very interesting to know whether the authors tested if other WAGOs, in particular SAGO-2, interact with TCER-1 or if the interaction is specific for PPW-1?

We apologize for the oversight in not noting the PA14 resistance phenotype of *sago-2* mutants, and have now included this in the revised manuscript (Pg. 12; Lines 219-221).

We attempted to assess the genetic interaction between SAGO-2 and TCER-1 using a combination of genetic mutants and RNAi, and obtained equivocal results as below.

In one trial, two *sago-2* mutants, *sago-2(tor135)* and *sago-2(tm894)*, survived significantly longer than wild-type controls, and exhibited no further increase in survival upon *tcer-1* RNAi. This suggests that, similar to PPW-1, SAGO-2 interacts genetically with TCER-1 to repress immunity.

However, in a second trial, while *tcer-1* RNAi did not extend the survival of *sago-2* mutants significantly, only the *sago-2(tm894)* in fact survived longer than WT and this was not statistically significant, so we could not deduce epistasis with accuracy.

Additionally*, sago-2* RNAi did not enhance survival of WT animals so we could not perform the reverse experiment (*sago-2* RNAi on *tcer-1* mutants) to assess the relationship. We were also unable to generate *tcer-1;sago-2* double mutants within a timeframe compatible with timely resubmission of the resubmitting the manuscript.

In light of these mixed findings, we chose not to include these data in the main figures. However, in the interest of transparency and completeness, we have discussed these findings in the text (Pg. 12; Lines 221-223) and included the data in a supplementary table (Table S9, below).

**Reviewer #3**

There are two issues which I think the authors should consider.

1) I'm puzzled about the tissue specificity of the response. It's not clear whether scrm-4 is expressed in intestine or germline and how this relates to the tissue specificity of the 22Gs that regulate it (which is explored earlier in the manuscript). It would be nice if the authors could do tissue specific RNAi of scrm-4 to see where it is required for pathogen resistance and relate this to the localisation of the particular wagos that regulate it.

We appreciate the reviewer's insightful suggestion. Unfortunately, tissue-specific RNAi of scrm-4 was not feasible in this study, as the strains required for such experiments (ppw-1, rrf-1, rde-1) themselves exhibit immune phenotypes that are characterized in this manuscript, precluding their use as clean genetic backgrounds. Similarly, we were unable to generate stable transcriptional reporter or overexpression strains that would have permitted a more thorough investigation of scrm-4 regulation and its site of action. Nevertheless, to address the question of tissue specificity, we interrogated several published RNA-seq and chromatin-mark datasets (detailed below), which collectively point to expression of scrm-4 in both germline and somatic tissues, as well as transcriptional regulation in both compartments.

2) It would be good to consider a little more the mode of regulation of scrm-4. Is this a post-transcriptional or a transcriptional (or both) silencing response? It seems to me, from a cursory glance at the genome browser, that scrm-4 may be in a repressive chromatin domain (H3K27me3 marked) which would imply that the regulation by wago may be transcriptional. Whilst it is probably beyond the scope of the manuscript to perform ChIP-seq experiments before and after infection and in the tcer-1 mutant, it would be nice if they could test genetically a requirement for nuclear RNAi, such as nrde-2 or nrde-3 (if scrm4 is somatic in this response, hrde-1 if it is germline). Moreover, it would be good for the authors to do some genome-browser snapshots to clearly show what is known about the baseline chromatin environment using, for example, the Ahringer lab datasets on chromatin domains.

We thank the reviewer for this suggestion. In the revised manuscript, we expanded our analysis of *scrm-4* regulation using several published genomics datasets. Briefly, RNA-seq data indicate that *scrm-4* is likely expressed weakly in both somatic and germline tissues. While the locus displays active chromatin marks (H3K4me3 and open chromatin by ATAC-seq), it is embedded within a broader H3K27me3-enriched domain with low H3K36me3, consistent with a repressive chromatin environment poised for regulated silencing. Furthermore, *scrm-4* is targeted by WAGO-associated 22G-RNAs that associate with both somatic cytoplasmic Argonautes (PPW-1/2 and WAGO-1) and the germline nuclear Argonaute, HRDE-1. As HRDE-1 is known to promote H3K9me3-mediated transcriptional silencing, these findings support a dual mechanism involving both post-transcriptional and transcriptional regulation. While genetic testing of nuclear RNAi factors such as *nrde-2* and *nrde-3* would be informative, the presence of HRDE-1-associated 22G-RNAs provides strong evidence for a nuclear component of this pathway. We have updated the manuscript to explicitly propose this dual-mode regulatory model and cite the Ahringer lab chromatin datasets used to characterize the genomic context of *scrm-4* (Pg.11, Lines 195-213). These data are shown below and added to the article as Fig. S2.

**Fig. S2.** **Expression and regulation of *scrm-4*. (A)** mRNA and sRNA read distribution across *scrm-4* and *tcer-1* in wildtype whole adult animals or dissected distal gonads. *mex-5* is shown as a germline-specific reference and *myo-3* as a soma-specific reference. One of three biological replicates is shown. Reads normalized by million mapped reads in each library. **(B)** Ahringer lab genome browser screenshot of ATAC- and CHIP-seq read distribution across the *scrm-4* locus. **(C)** Average log_2_ *scrm-4* 22G-RNA reads in various Argonaute coIPs relative to cell lysates. Reads were normalized by library size. n=2 biological replicates.

# Part III – Minor Points

**Reviewer #1**

1. I recommend that the authors include the paper (Kim and Han, PMID: 39414082) in the “Introduction” section to support the statement regarding gene silencing by miRNAs. In addition, I recommend that the authors add the article (Lee et al., PMID: 38614390) to provide accessible background information of RNA-sequencing, enabling readers to better follow the interpretation of the study.

The references have been added.

2. Please correct the significant errors and inconsistencies in the figure text.
   - In Abstract, line 2, please correct “Small interfering RNA (siRNA)” to “Small interfering RNAs (siRNAs)”.
   - On page 6, line 3, please correct “mut-16, mut-14 and smut-1” to “mut-16, mut-14, and smut-1”.
   - On page 6, line 10, please correct “for required” to “required for”.
   - On page 6, line 16, please correct “(Fig. 3C-D and Supplementary Table S4)” to “(Fig. 3C-D, Supplementary Table S4)”.

Corrected

3. I recommend that the authors provide full terms at their first appearance in the manuscript to enhance clarity.
   - On page 4, line 16, please provide full terms for “P. aeruginosa”.
   - On page 7, line 3, please provide full terms for “E. coli”.

Full forms have been included.

4. Please correct inconsistent space throughout the manuscript. Some examples are below:
   - On page 4, line 11, please correct the space between “resistance” and “[36]”.
   - On page 9, line 1, please correct the space between “ (Fig. 4D-E, Supplementary Tables S6-S7)” and “[58, 59]”.
   - On page 10, line 6, please correct the space between “as well” and “[23, 51, 65]”.
   - On page 11, line 22, please correct the space between “37” and “°C”.

Corrected

**Reviewer #2**

Survival data from additional trials are easy to find and clearly presented in supplementary tables, which is great, but raw data are missing (or I did not find them).

We thank the reviewer for this comment and appreciate the opportunity to clarify our data sharing approach.

All survival data in the article- mean survival numbers for every experiment and replicate trial- are provided in full within the paper and supplementary tables, and represent the complete quantitative basis for all analyses and conclusions. We are happy to make individual-level animal survival trajectories available to any interested party upon request. Two practical considerations inform this approach. First, the complete individual strain/treatment-level datasets spans more than 70 separate experimental files, making inclusion as supplementary material impractical. Second, PLOS policy states that authors are not required to submit raw data collected during an investigation "if the standard in the field is to share data that have been processed" (<https://journals.plos.org/plosone/s/data-availability>). In survival biology, mean lifespan values derived from replicate experiments are the standard unit of reported and shared data; individual animal-level death records are the underlying collection data from which these are derived, and it is not current field practice to make these routinely available. Generalist repositories such as Figshare or Dryad remain an option we are willing to pursue should the editor and reviewer strongly prefer formal deposition over an on-request policy.

We believe the data provided are sufficient to evaluate, reproduce, and build upon the reported findings, and we are fully committed to transparency. We have updated the Data Availability Statement to make our offer to share the complete individual-level survival data upon request explicit.

**Reviewer #3**

No minor points raised.
