# Supplementary material for: A TCER-1-siRNA regulatory axis suppresses antibacterial innate immunity in C. elegans
Source: PLoS Pathog. 2026 Jul 28;22(7):e1013972. doi: 10.1371/journal.ppat.1013972 (PMC13426946; doi:10.1371/journal.ppat.1013972)
Supplement: Peer review history

# Peer review: Decision Letter 0

**DOI:** 10.1371/journal.ppat.1013972.r001

**Authors:** Emily Troemel, D. Scott Samuels

11 Mar 2026

PPATHOGENS-D-26-00272

A TCER-1-siRNA Regulatory Axis Suppresses Antibacterial Innate Immunity in C. elegans

PLOS Pathogens

Dear Dr. Ghazi,

Thank you for submitting your manuscript to PLOS Pathogens. Your manuscript has been evaluated by members of the editorial board and three expert reviewers; all feel that the study is important and carefully implemented, but there are issues that you need to address before it can be published in PLOS Pathogens. Therefore, we invite you to submit a revised version of the manuscript that addresses the points raised during the review process.

Please submit your revised manuscript by May 10 2026 11:59PM. If you will need more time than this to complete your revisions, please reply to this message or contact the journal office at plospathogens@plos.org. When you're ready to submit your revision, log on to https://www.editorialmanager.com/ppathogens/ and select the 'Submissions Needing Revision' folder to locate your manuscript file.

Please include the following items when submitting your revised manuscript:

* A letter that responds to each point raised by the editor and reviewer(s). You should upload this letter as a separate file labeled 'Response to Reviewers'. This file does not need to include responses to any formatting updates and technical items listed in the 'Journal Requirements' section below.

* A marked-up copy of your manuscript that highlights changes made to the original version. You should upload this as a separate file labeled 'Revised Manuscript with Track Changes'.

* An unmarked version of your revised paper without tracked changes. You should upload this as a separate file labeled 'Manuscript'.

If you would like to make changes to your financial disclosure, competing interests statement, or data availability statement, please make these updates within the submission form at the time of resubmission. Guidelines for resubmitting your figure files are available below the reviewer comments at the end of this letter.

We look forward to receiving your revised manuscript.

Kind regards,

Emily R. Troemel

Academic Editor

PLOS Pathogens

D. Scott Samuels

Section Editor

PLOS Pathogens

Sumita Bhaduri-McIntosh

Editor-in-Chief

PLOS Pathogens

orcid.org/0000-0003-2946-9497

Michael Malim

Editor-in-Chief

PLOS Pathogens

orcid.org/0000-0002-7699-2064

**Journal Requirements:**

1) Please ensure that the CRediT author contributions listed for every co-author are completed accurately and in full.

At this stage, the following Authors/Authors require contributions: Spencer Kuhn. Please ensure that the full contributions of each author are acknowledged in the "Add/Edit/Remove Authors" section of our submission form.

The list of CRediT author contributions may be found here: https://journals.plos.org/plospathogens/s/authorship#loc-author-contributions

2) We ask that a manuscript source file is provided at Revision. Please upload your manuscript file as a .doc, .docx, .rtf or .tex. If you are providing a .tex file, please upload it under the item type u2018LaTeX Source Fileu2019 and leave your .pdf version as the item type u2018Manuscriptu2019.

3) Please provide an Author Summary. This should appear in your manuscript between the Abstract (if applicable) and the Introduction, and should be 150-200 words long. The aim should be to make your findings accessible to a wide audience that includes both scientists and non-scientists. Sample summaries can be found on our website under Submission Guidelines:

https://journals.plos.org/plospathogens/s/submission-guidelines#loc-parts-of-a-submission

4) Please amend your detailed Financial Disclosure statement. This is published with the article. It must therefore be completed in full sentences and contain the exact wording you wish to be published.

1) State what role the funders took in the study. If the funders had no role in your study, please state: "The funders had no role in study design, data collection and analysis, decision to publish, or preparation of the manuscript."

Note: If the reviewer comments include a recommendation to cite specific previously published works, please review and evaluate these publications to determine whether they are relevant and should be cited. There is no requirement to cite these works unless the editor has indicated otherwise.

**Reviewers' Comments:**

Reviewer's Responses to Questions

**Part I - Summary**

Please use this section to discuss strengths/weaknesses of study, novelty/significance, general execution and scholarship.

Reviewer #1: In this manuscript entitled “A TCER-1-siRNA Regulatory Axis Suppresses Antibacterial Innate Immunity in C. elegans,” the authors investigated how endogenous siRNA pathways modulate antibacterial immunity against Pseudomonas aeruginosa PA14. The authors identified endogenous small RNAs, WAGO Argonaute-associated 22G-RNAs, that repress antibacterial immunity. The authors found that multiple components required for WAGO 22G-RNA biogenesis suppress host resistance through TCER-1, which is transcription elongation and splicing factor. They also showed that TCER-1 targets a restricted set of immune-relevant effectors, such as scrm-4, rather than inducing widespread gene silencing. The authors highlighted a TCER-1-siRNA regulatory axis in which TCER-1 promotes WAGO 22G-RNA biogenesis, thereby repressing antibacterial immunity.

Reviewer #2: In this manuscript, Naim and colleagues provide new insights into the role of small interfering RNAs (siRNAs) in C. elegans antibacterial defense. They demonstrate that TCER-1, the C. elegans homolog of human transcription elongation and splicing factor TCERG1, suppresses host defense against Pseudomonas aeruginosa PA14 via the WAGO 22G-RNA pathway. The role of TCER-1 as a repressor of host defense against bacterial pathogens had been established in previous work by the same group. High-throughput small RNA and mRNA sequencing data from two tcer-1 mutants revealed that expression of WAGO 22G-RNA pathway genes was not down-regulated in tcer-1 mutants. Thus, TCER-1 probably does not directly affect the WAGO 22G-RNA pathway, but rather indirectly influences stability of 22G-RNAs or the processing of WAGO targets. The exact mechanism of the interaction remains unclear. However, the authors also find that the WAGO target scrm-4, which encodes a phospholipid translocase, contributes to pathogen defenses in a TCER-1-dependent manner.

These results are significant and novel. While the role of miRNAs in antibacterial host defense in Caenorhabditis elegans is well established, endogenous siRNAs have been primarily studied in the context of antiviral defense. Although emerging evidence suggests that endogenous siRNA pathways may influence antibacterial immunity, their precise contribution to antibacterial host defense remains unclear. This study provides direct evidence of endogenous siRNA participation in antibacterial pathways, via interaction with TCER-1 and the WAGO target gene scrm-4. It significantly broadens our understanding of the role of endo-siRNAs in host-pathogen interactions and is thus of high importance and broad interest to the community in this field.

The manuscript is clearly written, and the experiments have been performed rigorously and are presented in a convincing and comprehensive manner. I only have two comments, including a suggestion for an additional experiment. Other than that, publication of the presented results as they stand is in my opinion justified.

Reviewer #3: In this manuscript the authors link the 22GRNA pathway in C. elegans to pathogen resistance, via the factor TCER-1 which acts upstream of 22G pathways. They provide convincing evidence for the alteration of 22G-RNAs against a small number of genes when TCER-1 is inactive and, nicely, are able to trace the effect on pathogen resistance to a particular target, scrm-4. The genetics experiments, statistical and computational analyses are excellent and the paper is written clearly and logically, without overstating the results.

**********

**Part II – Major Issues: Key Experiments Required for Acceptance**

Please use this section to detail the key new experiments or modifications of existing experiments that should be absolutely required to validate study conclusions.

Generally, there should be no more than 3 such required experiments or major modifications for a "Major Revision" recommendation. If more than 3 experiments are necessary to validate the study conclusions, then you are encouraged to recommend "Reject".

Reviewer #1: 1. In Figure 1E, the authors conclude that TCER-1::GFP expression is unchanged by ppw-1 or rrf-1 mutations, but the evidence is presented mainly as representative images. I recommend that the authors include a quantitative analysis of GFP expression from the TCER-1::GFP translation reporter. This would strengthen the conclusion that ppw-1 or rrf-1 mutations do not alter TCER-1 expression.

2. Fourteen targets show strong anti-correlation between 22G-RNAs and mRNA levels, and three, including scrm-4, exhibit changes greater than four-fold. It remains unclear why scrm-4 was prioritized over the others. Please write the justification for that. The epistasis data indicate that scrm-4 is required for tcer-1 RNAi-mediated immunoresistance. Can they add similar experimental results using WAGO mutants? Overexpression study will also strengthen the role of scrm-4 as a downstream target. If the authors do not have such genetric tools for testing these, it will be nice to add discussion points regarding this issue.

Reviewer #2: The study focuses on WAGO Argonaute-associated siRNAs and is based on the intriguing observation that the ppw-1 mutant of the WAGO 22G-RNA pathway, which is used in C. elegans for germline- vs. soma-specific gene-knockdown by RNAi, exhibited clearly decreased susceptibility to infection with P. aeruginosa PA14. PPW-1 is a member of the WAGO clade in C. elegans. Of the 13 AGOs in the WAGO clade, SAGO-2 and PPW-1 were previously shown to be involved in defense against PA14 infection (both sago-2 and ppw-1 mutants are resistant to PA14 infection; Seroussi et al., 2023 https://doi.org/10.7554/eLife.83853). Therefore, it would be very interesting to know whether the authors tested if other WAGOs, in particular SAGO-2, interact with TCER-1 or if the interaction is specific for PPW-1?

Reviewer #3: There are two issues which I think the authors should consider.

1) I'm puzzled about the tissue specificity of the response. It's not clear whether scrm-4 is expressed in intestine or germline and how this relates to the tissue specificity of the 22Gs that regulate it (which is explored earlier in the manuscript). It would be nice if the authors could do tissue specific RNAi of scrm-4 to see where it is required for pathogen resistance and relate this to the localisation of the particular wagos that regulate it.

2) It would be good to consider a little more the mode of regulation of scrm-4. Is this a post-transcriptional or a transcriptional (or both) silencing response? It seems to me, from a cursory glance at the genome browser, that scrm-4 may be in a repressive chromatin domain (H3K27me3 marked) which would imply that the regulation by wago may be transcriptional. Whilst it is probably beyond the scope of the manuscript to perform ChIP-seq experiments before and after infection and in the tcer-1 mutant, it would be nice if they could test genetically a requirement for nuclear RNAi, such as nrde-2 or nrde-3 (if scrm4 is somatic in this response, hrde-1 if it is germline). Moreover, it would be good for the authors to do some genome-browser snapshots to clearly show what is known about the baseline chromatin environment using, for example, the Ahringer lab datasets on chromatin domains.

**********

**Part III – Minor Issues: Editorial and Data Presentation Modifications**

Please use this section for editorial suggestions as well as relatively minor modifications of existing data that would enhance clarity.

Reviewer #1: 1. I recommend that the authors include the paper (Kim and Han, PMID: 39414082) in the “Introduction” section to support the statement regarding gene silencing by miRNAs. In addition, I recommend that the authors add the article (Lee et al., PMID: 38614390) to provide accessible background information of RNA-sequencing, enabling readers to better follow the interpretation of the study.

2. Please correct the significant errors and inconsistencies in the figure text.

- In Abstract, line 2, please correct “Small interfering RNA (siRNA)” to “Small interfering RNAs (siRNAs)”.

- On page 6, line 3, please correct “mut-16, mut-14 and smut-1” to “mut-16, mut-14, and smut-1”.

- On page 6, line 10, please correct “for required” to “required for”.

- On page 6, line 16, please correct “(Fig. 3C-D and Supplementary Table S4)” to “(Fig. 3C-D, Supplementary Table S4)”.

3. I recommend that the authors provide full terms at their first appearance in the manuscript to enhance clarity.

- On page 4, line 16, please provide full terms for “P. aeruginosa”.

- On page 7, line 3, please provide full terms for “E. coli”.

4. Please correct inconsistent space throughout the manuscript. Some examples are below:

- On page 4, line 11, please correct the space between “resistance” and “[36]”.

- On page 9, line 1, please correct the space between “ (Fig. 4D-E, Supplementary Tables S6-S7)” and “[58, 59]”.

- On page 10, line 6, please correct the space between “as well” and “[23, 51, 65]”.

- On page 11, line 22, please correct the space between “37” and “°C”.

Reviewer #2: Survival data from additional trials are easy to find and clearly presented in supplementary tables, which is great, but raw data are missing (or I did not find them).

Reviewer #3: (No Response)

**********

PLOS authors have the option to publish the peer review history of their article (what does this mean?). If published, this will include your full peer review and any attached files.

If you choose “no”, your identity will remain anonymous but your review may still be made public.

**Do you want your identity to be public for this peer review?**  For information about this choice, including consent withdrawal, please see our Privacy Policy.

Reviewer #1: No

Reviewer #2: No

Reviewer #3: No

[NOTE: If reviewer comments were submitted as an attachment file, they will be attached to this email and accessible via the submission site. Please log into your account, locate the manuscript record, and check for the action link "View Attachments". If this link does not appear, there are no attachment files.]

**Figure resubmission:**

-->While revising your submission, we strongly recommend that you use PLOS’s NAAS tool (https://ngplosjournals.pagemajik.ai/artanalysis) to test your figure files. NAAS can convert your figure files to the TIFF file type and meet basic requirements (such as print size, resolution), or provide you with a report on issues that do not meet our requirements and that NAAS cannot fix.

After uploading your figures to PLOS’s NAAS tool - https://ngplosjournals.pagemajik.ai/artanalysis, NAAS will process the files provided and display the results in the "Uploaded Files" section of the page as the processing is complete. If the uploaded figures meet our requirements (or NAAS is able to fix the files to meet our requirements), the figure will be marked as "fixed" above. If NAAS is unable to fix the files, a red "failed" label will appear above. When NAAS has confirmed that the figure files meet our requirements, please download the file via the download option, and include these NAAS processed figure files when submitting your revised manuscript.

**Reproducibility:**

To enhance the reproducibility of your results, we recommend that authors of applicable studies deposit laboratory protocols in protocols.io, where a protocol can be assigned its own identifier (DOI) such that it can be cited independently in the future. Additionally, PLOS ONE offers an option to publish peer-reviewed clinical study protocols. Read more information on sharing protocols at https://plos.org/protocols?utm_medium=editorial-email&utm_source=authorletters&utm_campaign=protocols

# Author comment: Author response to Decision Letter 1

**DOI:** 10.1371/journal.ppat.1013972.r002

26 Jun 2026

Attachment Submitted filename: RESP_REVIEWERS_23JUN26_NaimEtal_FINAL.docx

# Peer review: Decision Letter 1

**DOI:** 10.1371/journal.ppat.1013972.r003

**Authors:** Emily Troemel, D. Scott Samuels

6 Jul 2026

Dear Dr. Ghazi,

We are pleased to inform you that your manuscript 'A TCER-1-siRNA Regulatory Axis Suppresses Antibacterial Innate Immunity in C. elegans' has been provisionally accepted for publication in PLOS Pathogens.

Before your manuscript can be formally accepted you will need to complete some formatting changes, which you will receive in a follow up email. A member of our team will be in touch with a set of requests.

Please note that your manuscript will not be scheduled for publication until you have made the required changes, so a swift response is appreciated.

IMPORTANT: The editorial review process is now complete. PLOS will only permit corrections to spelling, formatting or significant scientific errors from this point onwards. Requests for major changes, or any which affect the scientific understanding of your work, will cause delays to the publication date of your manuscript.

Should you, your institution's press office or the journal office choose to press release your paper, you will automatically be opted out of early publication. We ask that you notify us now if you or your institution is planning to press release the article. All press must be co-ordinated with PLOS.

Thank you again for supporting Open Access publishing; we are looking forward to publishing your work in PLOS Pathogens.

Best regards,

Emily R. Troemel

Academic Editor

PLOS Pathogens

D. Scott Samuels

Section Editor

PLOS Pathogens

Sumita Bhaduri-McIntosh

Editor-in-Chief

PLOS Pathogens

orcid.org/0000-0003-2946-9497

Michael Malim

Editor-in-Chief

PLOS Pathogens

orcid.org/0000-0002-7699-2064

***********************************************************

Reviewer Comments (if any, and for reference):

Reviewer's Responses to Questions

**Part I - Summary**

Please use this section to discuss strengths/weaknesses of study, novelty/significance, general execution and scholarship.

Reviewer #1: The authors addressed my concerns successfully.

Reviewer #2: The authors have satisfactorily addressed my comments, as well as the majority of the concerns raised by the other reviewers, resulting in a further improvement of the manuscript. I have no additional comments.

Reviewer #3: As in first review

**********

**Part II – Major Issues: Key Experiments Required for Acceptance**

Please use this section to detail the key new experiments or modifications of existing experiments that should be absolutely required to validate study conclusions.

Generally, there should be no more than 3 such required experiments or major modifications for a "Major Revision" recommendation. If more than 3 experiments are necessary to validate the study conclusions, then you are encouraged to recommend "Reject".

Reviewer #1: (No Response)

Reviewer #2: (No Response)

Reviewer #3: No remaining major issues- the authors have done a really nice job of addressing the comments that I made previously

**********

**Part III – Minor Issues: Editorial and Data Presentation Modifications**

Please use this section for editorial suggestions as well as relatively minor modifications of existing data that would enhance clarity.

Reviewer #1: (No Response)

Reviewer #2: (No Response)

Reviewer #3: No minor issues

**********

PLOS authors have the option to publish the peer review history of their article (what does this mean?). If published, this will include your full peer review and any attached files.

If you choose “no”, your identity will remain anonymous but your review may still be made public.

**Do you want your identity to be public for this peer review?**  For information about this choice, including consent withdrawal, please see our Privacy Policy.

Reviewer #1: No

Reviewer #2: No

Reviewer #3: **Yes:**  Peter Sarkies

# Editor report: Acceptance letter

**DOI:** 10.1371/journal.ppat.1013972.r004

**Authors:** Emily Troemel, D. Scott Samuels

Dear Dr. Ghazi,

We are delighted to inform you that your manuscript, "A TCER-1-siRNA Regulatory Axis Suppresses Antibacterial Innate Immunity in C. elegans," has been formally accepted for publication in PLOS Pathogens.

We have now passed your article onto the PLOS Production Department who will complete the rest of the pre-publication process. All authors will receive a confirmation email upon publication.

The corresponding author will soon be receiving a typeset proof for review, to ensure errors have not been introduced during production. Please review the PDF proof of your manuscript carefully, as this is the last chance to correct any scientific or type-setting errors. Please note that major changes, or those which affect the scientific understanding of the work, will likely cause delays to the publication date of your manuscript. Note: Proofs for Front Matter articles (Pearls, Reviews, Opinions, etc...) are generated on a different schedule and may not be made available as quickly.

Soon after your final files are uploaded, the early version of your manuscript, if you opted to have an early version of your article, will be published online. The date of the early version will be your article's publication date. The final article will be published to the same URL, and all versions of the paper will be accessible to readers.

For Research Articles, you will receive an invoice from PLOS for your publication fee after your manuscript has reached the completed accept phase. If you receive an email requesting payment before acceptance or for any other service, this may be a phishing scheme. Learn how to identify phishing emails and protect your accounts at https://explore.plos.org/phishing.

Thank you again for supporting open-access publishing; we are looking forward to publishing your work in PLOS Pathogens.

Best regards,

Sumita Bhaduri-McIntosh

Editor-in-Chief

PLOS Pathogens

orcid.org/0000-0003-2946-9497

Michael Malim

Editor-in-Chief

PLOS Pathogens

orcid.org/0000-0002-7699-2064
